# Study on the material source and enrichment mechanism of REE-rich phosphorite in Zhijin, Guizhou

**DOI:** 10.1038/s41598-024-57074-2

**Published:** 2024-03-18

**Authors:** Jingya Wang, Zhongkun Qiao

**Affiliations:** Institute of Frontiers and Interdisciplinary Sciences, Zhejiang University of Technology, No. 18 Chaowang Road, Hangzhou, 310014 Zhejiang Province China

**Keywords:** Rare earth elements, Marine sedimentary phosphorite, Geochemical characteristics, Material sources, Sedimentary environments, Ocean sciences, Solid Earth sciences

## Abstract

Rare earth element (REE)-rich phosphorite in the Guizhou region mainly exists in the Doushantuo Formation and Gezhongwu Formation in early Cambrian strata, which are some of the important strata containing phosphorite resources in China. The early Cambrian Zhijin phosphorite in Guizhou Province, China, has high rare earth element and yttrium (REY) contents of up to 2500 ppm, with heavy REY (HREY) contents accounting for ~ 30% of the total REY contents. However, the specific controlling source and environment of phosphorite (especially the REEs in Zhijin phosphorite) are still unsolved. Through field geological investigations; mineralogical, geochemical, Sr–Nd isotope analyses; and tectonic characteristics, the material source, sedimentary environment and seawater dynamics of REEs in phosphorite are studied. It is considered that the REEs enriched in the Zhijin phosphorite are mainly affected by precipitation from hydrothermal fluid. Moreover, from the late Ediacaran to the early Cambrian, the depositional environment from the bottom to the top of the water tended to be hypoxic, and the activity of hot water fluid tended to be strong. The change in redox conditions is closely related to the rise and fall of sea level. Combined with the tectonic background, these results show that the weakly oxidized environment may be an important factor controlling the enrichment of REEs. The enrichment of REEs may be closely related to volcanic hydrothermal activity, later diagenesis and seawater dynamics.

## Introduction

Rare earth elements (REEs) refer to the lanthanide family with atomic numbers of 57–71. Scandium and yttrium in Group IIB are also usually included because of their similar geochemical characteristics^1^. Since their initial discovery and extraction, the uses of REEs have changed from rare earth mischmetal used in lighter flints to high-purity separated rare earth metals being used in advanced electronics, lighting, power generation and military applications^2^. At present, most of the world's REEs are produced from the Bayan Obo carbonate-type REE deposit and the ion-adsorption-type REE deposit in China^3^. The sharp decline in China's REE reserves has placed great demand on the exploration of new REE deposits (and/or types of REE deposits) for the sustainable development of global REE resources.

In recent years, attention has been focused on shallow marine sedimentary phosphorite deposits that are often associated with high REE contents. The highest REE contents ever observed in the Late Ordovician phosphorites in Arkansas and in the Late Devonian deposits in Kentucky reach 6878 ppm and 13,385 ppm, respectively^4^. In contrast, the REE content in phosphorite deposits in China is also quite high. In the Guizhou Zhijin phosphorite deposit, which is a typical REE-rich phosphorite deposit that formed in the early Cambrian, the average REE content reaches 2500 ppm^5,6^. REE-rich phosphorite deposits in shallow marine sedimentary rocks are thus expected to become the next potential REE resources to resolve the global shortage of REEs^7^.

At present, phosphorite deposits have been discovered to have formed in the Cambrian and later geological times. However, not all shallow sedimentary phosphorites contain a high content of REEs, and the REE content in the shallow sedimentary phosphorites seems to be highly variable, random and irregular. For example, both the Weng'an phosphorite deposits in Guizhou, China, which formed in the Ediacaran, and the Kunyang phosphorite deposits in Yunnan (the neighboring province of Guizhou), which formed in the Cambrian, contain low REE contents^8,9^. In contrast, the Zhijin phosphorite, which is located in the same area as the Weng'an phosphorite deposits and formed in the same geological period as the Kunyang phosphorite deposits, has been reported to have a high REE content^10^. Moreover, their REE post-Archean Australian shale (PAAS)-normalized patterns are significantly different^8,10^.

Furthermore, the material sources and enrichment mechanism of REEs in phosphorite are more controversial. It is generally believed that the content of REEs is positively related to the phosphorus content and that REEs are homologous to phosphorus^6,11^. Yang et al.^12^ believed that REE enrichment is mainly controlled by the REE content in the sedimentary environment and deep water. According to the relationship between phosphorus and organisms, Gao et al.^13^ believed that living organisms and dead remains can ingest and accumulate REEs in water to varying degrees. Shi et al.^14^ and Guo et al.^6^ believed that REEs may be affected by weathering and hydrothermal activities. Ilyin et al.^15^ believed that the REE contents of phosphorites are influenced by seawater compositions during geological history, while other researchers suggested that the enrichment of REEs may be caused by diagenesis^16–18^. As the material source and enrichment mechanism of REEs are not clear, the study of rare earth resources will be constrained.

In view of this, the study sought to determine the controlling material source and enrichment mechanism of REEs in phosphorite. We analyzed samples collected from the Zhijin and Weng’an phosphorite deposits, with their sedimentary environment, petrographic characteristics, geochemistry, and diagenetic process as the research content. This study provides a theoretical reference for exploring REEs in phosphorite.

## Background

**Figure 1:**
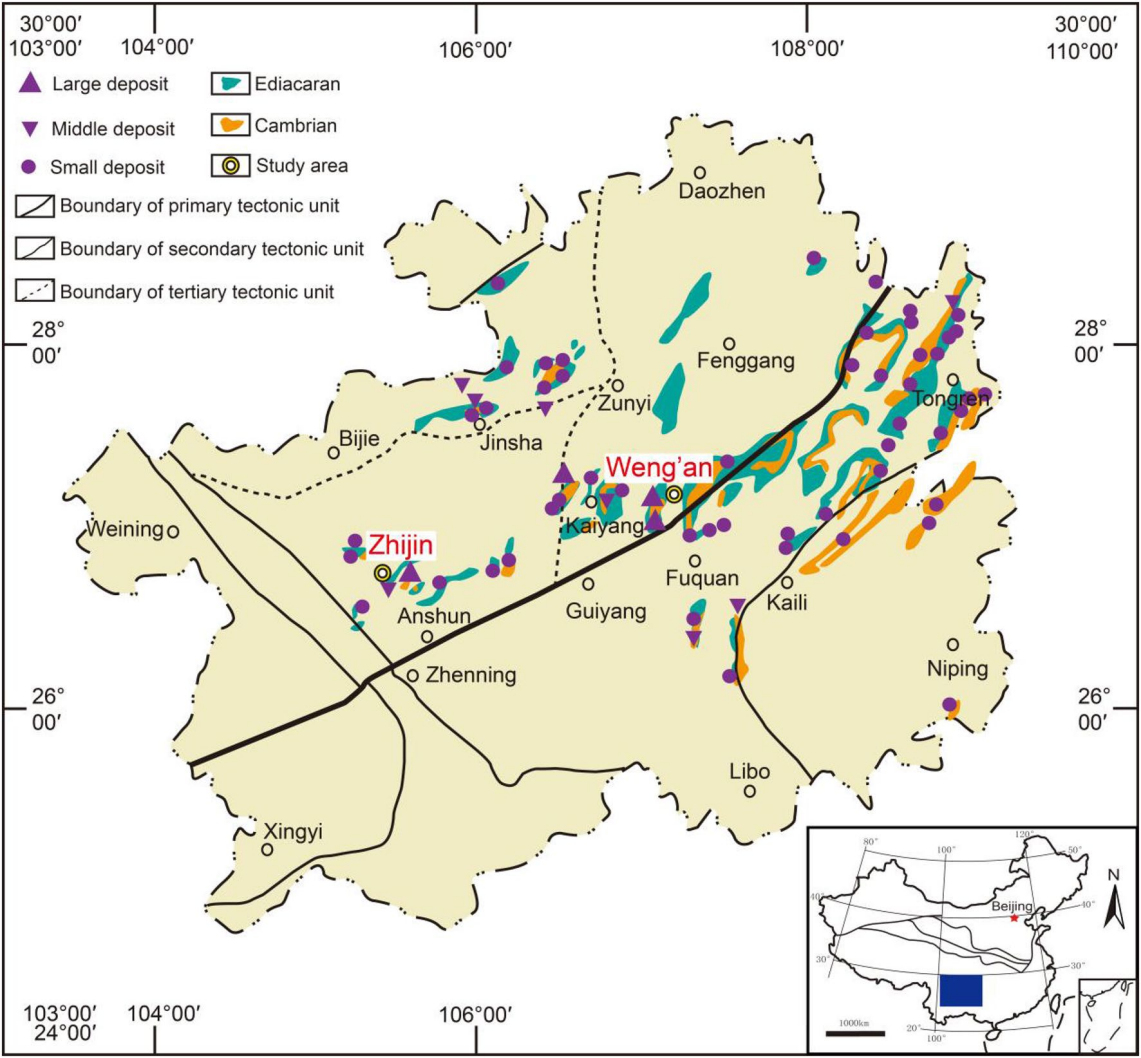
Tectonic sketch map and phosphorite distributions in Guizhou^19^ (modified from reference^19^ and created it with Adobe Illustrator CS6. http://www.adobe.com/products/illustrator.html).

**Figure 2:**
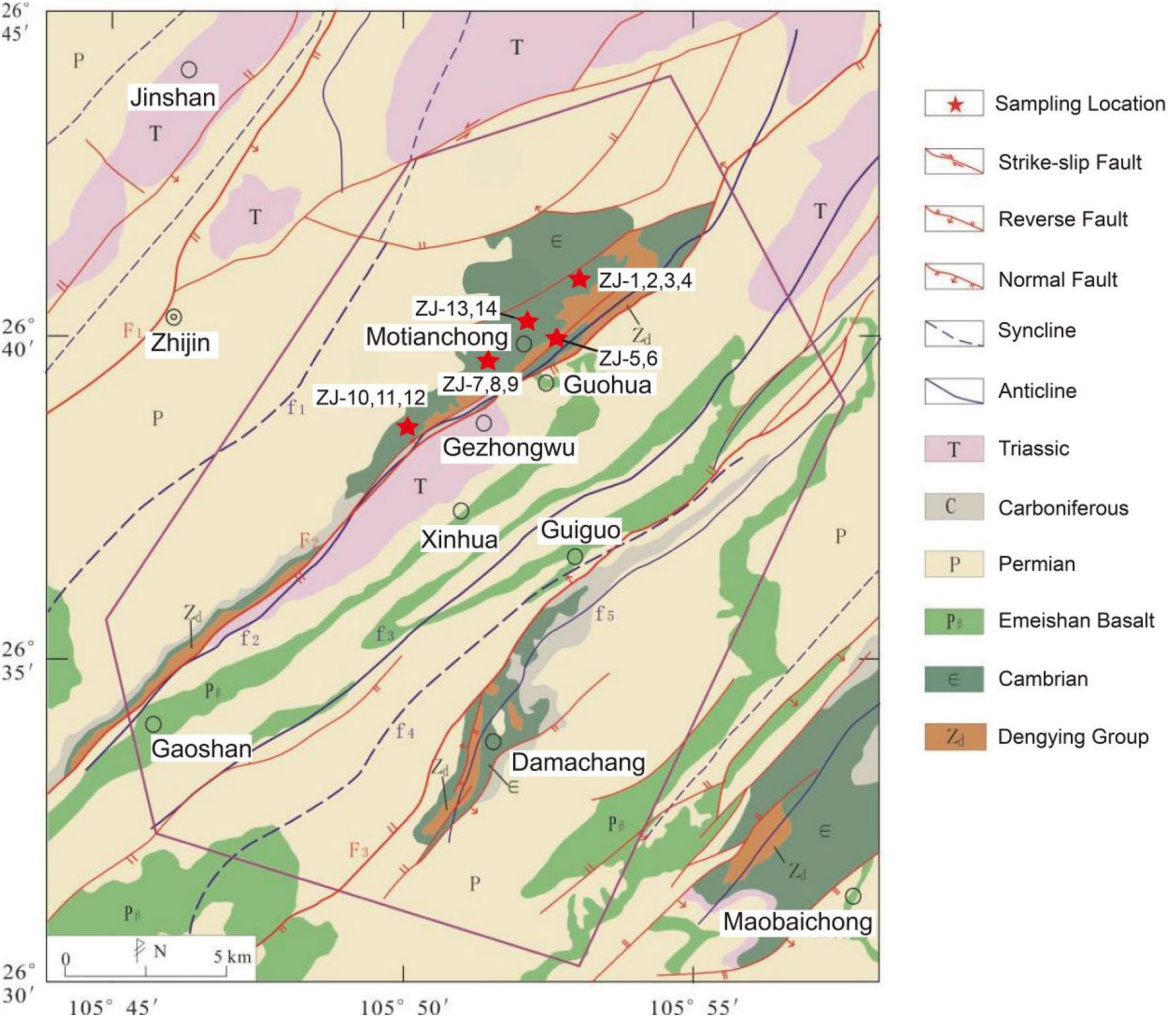
The tectonic geological picture of Zhijin phosphorite deposit^20^ (modified from reference^20^ and created it with Adobe Illustrator CS6. http://www.adobe.com/products/illustrator.html).

**Figure 3:**
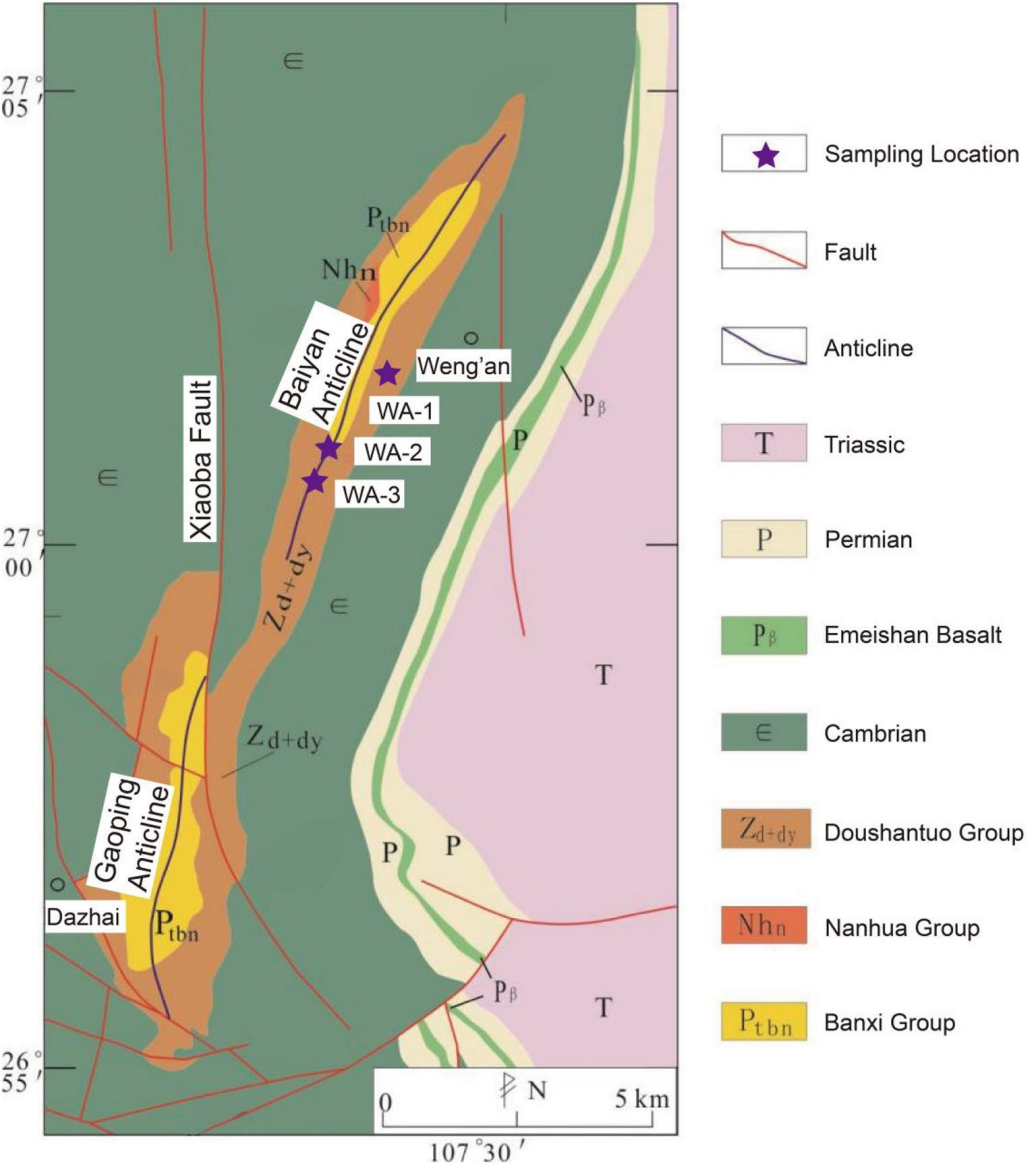
The tectonic geological picture of Weng’an phosphorite deposit^21^ (modified from reference^21^ and created it with Adobe Illustrator CS6. http://www.adobe.com/products/illustrator.html).

Regionally, Guizhou Province is part of the Yangtze plate, which is mainly composed of phosphorite from the Ediacaran Doushantuo Formation and Cambrian Gezhongwu Formation. The large phosphorite deposits are mainly distributed in Weng'an, Fuquan, Kaiyang, and Zhijin. In addition, medium-sized phosphorite deposits are distributed in Songtao, Jinsha, Duyun, Zunyi and other areas (Fig. 1). According to the new regional geological map of Guizhou Province, the typical phosphorite of the Cambrian Gezhongwu Formation is located in southeastern Zhijin County, Guizhou Province (Fig. 2). The typical phosphorite of the Ediacaran Doushantuo Formation is located in Weng'an, Guizhou Province (Fig. 3).

The tectonic lines (including faults and folds) in the Zhijin phosphorite deposit are mainly spread from north to east, and the tectonic deformation is mainly dominated by positive faults, reverse faults, and strike-slip faults (Fig. 2). Among them, the Gezhongwu phosphorite area is controlled by the northeast Guohua-Gezhongwu fault, which makes the Cambrian formation spread out along the fault, consistent with the fault and anticline direction. The Damachang phosphorite area is controlled by the Guiguo-Damachang fault. The Maobaichong phosphorite area is controlled by a series of northeast faults. The strata in the Zhijin phosphorite deposit are Ediacaran (Z), Cambrian (ε), Devonian (D), Carboniferous (C), Permian (P), and Triassic (T) (Fig. 2). Among them, the Cambrian Gezhongwu Formation is the formation bearing REE-rich phosphorite. The lower ore layer is composed of dolomaceous phosphorite and phospho-dolomite, which are rich in microorganisms. The upper ore layer is phospho-claystone and siltstone. The Gezhongwu Formation unconformably contacts the underlying Dengying Formation, and the overlying strata are carbonaceous shales of the Niutitang Formation^19^.

The Weng'an phosphorite deposit in Guizhou is located in the southeastern margin of the Yangtze Craton. The phosphorite originates from the nucleus and wing of the Baiyan-Gaoping anticline. The anticline saddle part is truncated by the north‒south Xiaoba fault, with the Baiyan anticline in the north and the Gaoping anticline in the south. Both the anticlines and the Weng'an phosphorite deposit are controlled by the Xiaoba fracture. The strata in the Weng'an phosphorite are Ediacaran (Z), Cambrian (ε), Permian (P), Triassic (T) and Jurassic (J) (Fig. 3). Among them, the late Ediacaran Doushantuo Formation is the formation bearing phosphorite. It is in false contact with the underlying Nantuo Formation and conforms to the overlying Dengying Formation. The lower ore layer is sandstone phosphorite, and the upper ore layer is dolomaceous phosphorite^19^.

## Samples and methods

### Sample collection

**Figure 4:**
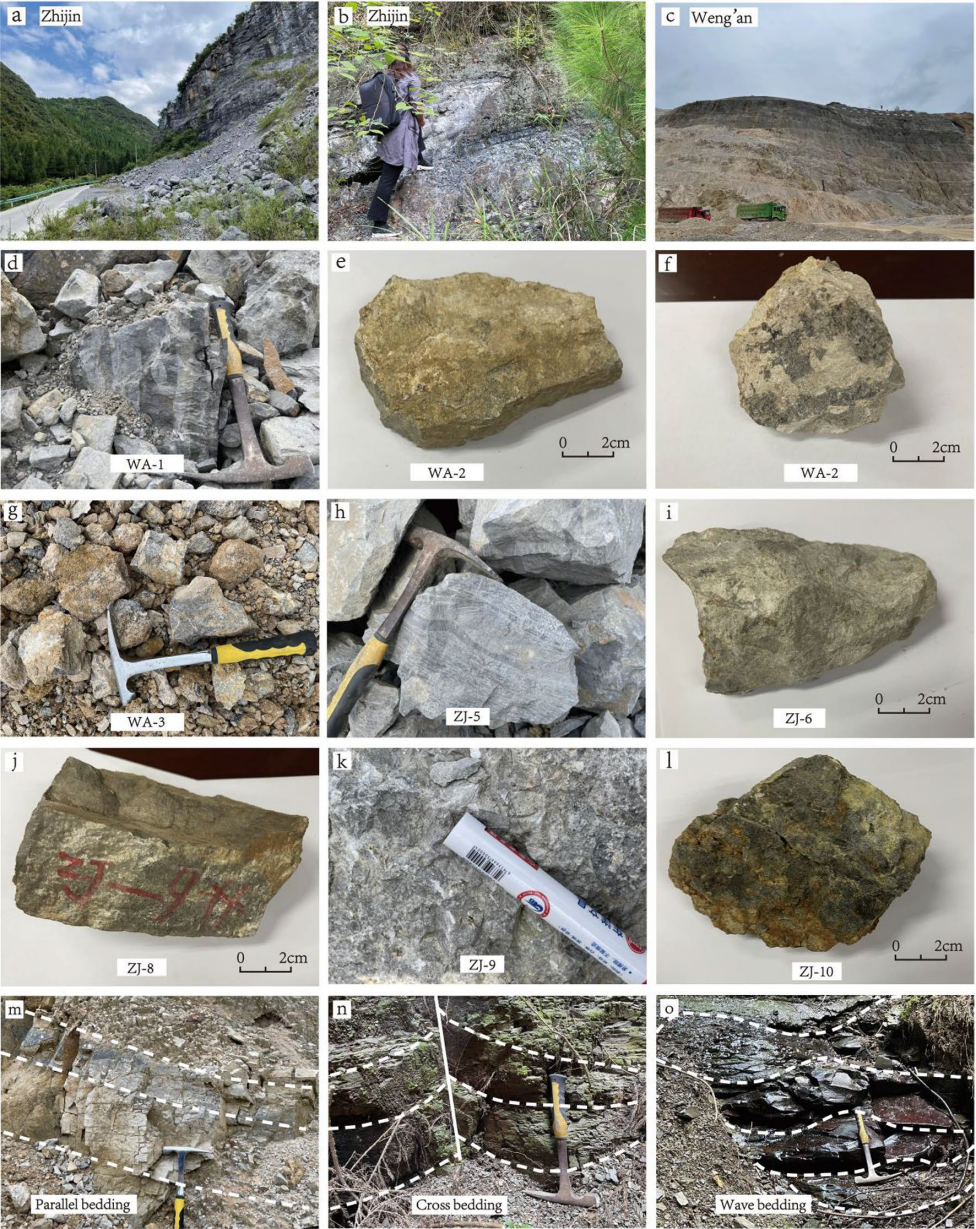
The field structural features of phosphorite from Gezhongwu and Weng’an phosphorite deposits. (**a**) Zhijin outcrops. (**b**) Zhijin outcrops. (**c**) Weng’an outcrops. (**d**) Banding structure. (**e**) Massive structure. (**f**) Breccia structure. (**g**) Earthy structure. (**h**) Laminar structure. (**i**) Clumpy structure. (**j**) Cataclastic structure. (**k**) Crumb structure. (**l**) Spherical structure. (**m**) Parallel bedding. (**n**) Cross bedding. (**o**) Wave bedding.

Fourteen samples were collected from the Gezhongwu Formation of the Zhijin phosphorite deposit (Fig. 4a–c). For comparison, three additional samples were collected from the Doushantuo Formation of the Weng’an phosphorite deposit. Sample features are shown in Table 1.

**Table 1:** Features of samples from the Zhijin and Weng’an phosphorite deposits.

No	Mining area	Stratum	Lithology	Color
ZJ-1	Zhijin phosphorite deposit	Dengying formation	Dolomite	Grayish white
ZJ-2			Dolomite	Grayish white
ZJ-3			Dolomite	Grayish white
ZJ-4			Dolomite	Grayish white
ZJ-5		Gezhongwu formation	Phosphorite	Dark gray
ZJ-6			Phosphorite	Dark gray
ZJ-7			Phosphorite	Dark gray
ZJ-8			Phosphorite	Dark gray
ZJ-9			Phosphorite	Dark gray
ZJ-10			Phosphorite	Dark gray
ZJ-11		Niutitang formation	Carbonaceous Shale	Dark brown
ZJ-12			Carbonaceous Shale	Dark brown
ZJ-13			Carbonaceous Shale	Dark brown
ZJ-14			Carbonaceous Shale	Dark brown
WA-1	Weng’an phosphorite deposit	Doushantuo formation	Phosphorite	Pale
WA-2			Phosphorite	Grayish yellow
WA-3			Phosphorite	Grayish yellow

### Petrographic analysis

The structures in phosphorites are banding, lamination, massive, brecciated, spherical, cataclastic, clumpy and earthy. Among them, the structures in phosphorites in the Doushantuo Formation are mainly banding (Fig. 4d), massive (Fig. 4e), brecciated (Fig. 4f), and earthy (Fig. 4g). Banding is the most common structure in phosphorite, with a regular dark gray and light white pattern and a high ore grade. The massive structure is brownish yellow, dense and hard. The mineral particles are evenly distributed, undeveloped or rarely intermittent in dolomite layers. The brecciated structure is dark gray breccia cemented by light gray dolomite, with particle sizes of 2–3 mm, indicating that the primary phosphorite was broken and cemented by dolomite after migration. The earthy structure is earth-yellow, fragile and soft. This structure is always found in weathered phosphorite.

The structures in phosphorites in the Gezhongwu Formation are mainly laminations (Fig. 4h), followed by clumpy (Fig. 4i), cataclastic (Fig. 4j,k) and spherical structures (Fig. 4l). The laminations are intermittently developed with flocculent, brecciated and clumped structures interspersed in dolomite, and a few layers have thicknesses of less than 1 mm. The clumpy structure is an ellipsoid with particle sizes of 3–5 cm. The clumps are pure phosphorite and quartz. The cataclastic structure shows irregular breccia with particle sizes of 2–5 mm and is filled by dolomite veins. The spherical structure is spherical, with gravel and sandstone phosphorite cemented by dolomite, and the particle sizes of sand are 3–5 mm.

Parallel bedding is present in the Doushantuo Formation phosphorites of Weng'an (Fig. 4m), while parallel bedding and cross-bedding are present in the Gezhongwu Formation phosphorites of the Zhijin deposit (Fig. 4n). Wave bedding is present in some areas of the Gezhongwu Formation phosphorites (Fig. 4o).

This shows that the Zhijin phosphorites were subjected to stronger seawater dynamic action in the process of sedimentation. The particle size of the Zhijin Gezhongwu Formation phosphorites is larger than that of the Weng 'an Doushantuo Formation, and the structural maturity of the Zhijin Gezhongwu Formation phosphorites is higher. Compared with Weng'an phosphorites, Zhijin phosphorites were subjected to greater mechanical power from seawater, and the seawater depth was shallower.

Microscopic rock and mineral identification was completed in the Earth Science Laboratory of Zhejiang University, with a magnification of 5–50 times. The mineral composition and texture of the rocks were observed under single polarized light, orthogonally polarized light and reflected light. Phosphorite from the Zhijin and Weng’an deposits consists of phosphate, dolomite, feldspar, quartz, calcite, mica and other minerals. The mineral texture mainly includes internal clastic and bioclastic structures and amorphous collophanite.

**Figure 5:**
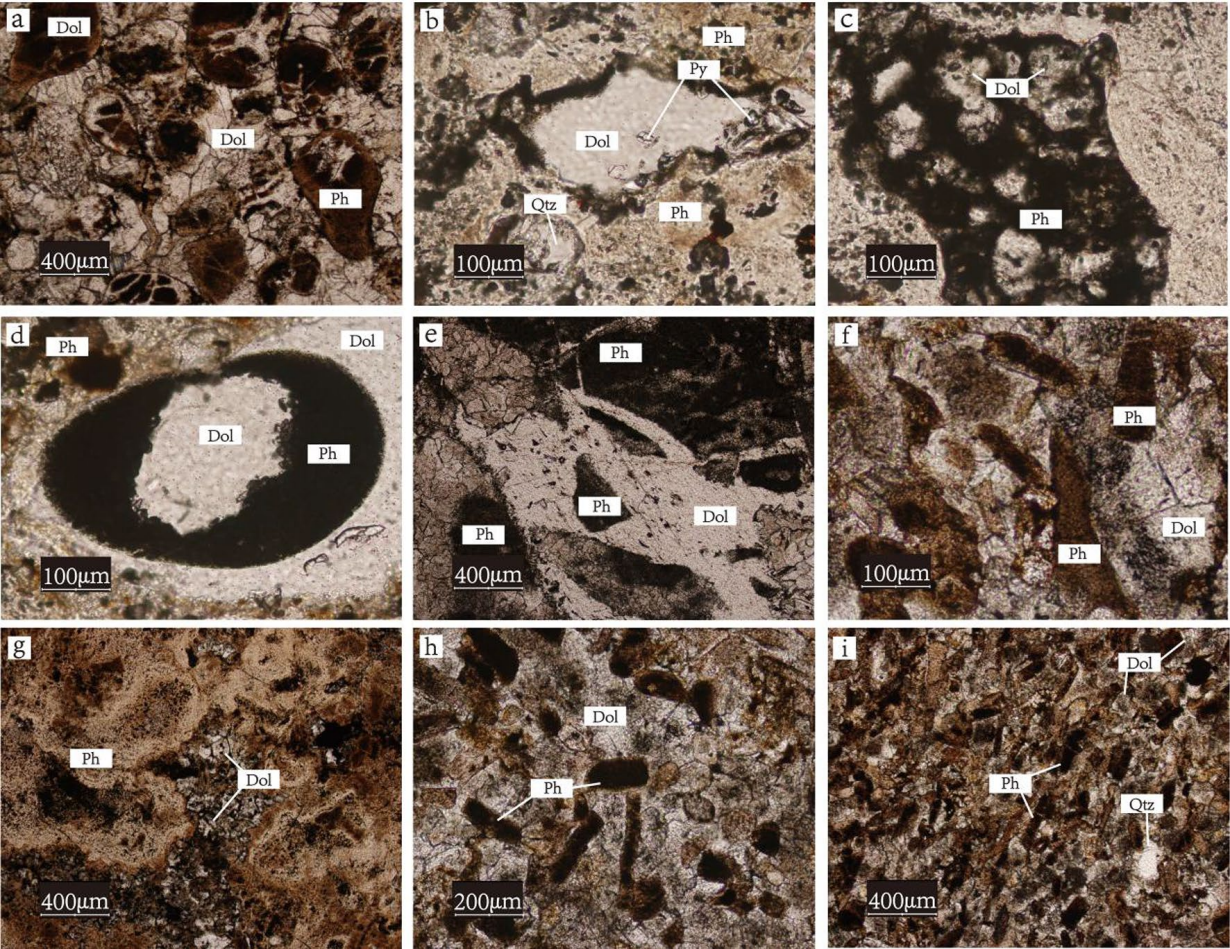
The microstructural features of phosphorite from Gezhongwu and Weng’an phosphorite deposits. Ph-phosphate; Dol-dolomite; Qtz-quartz; Py-pyrite.

Mineralogical characteristics show that Weng'an phosphorite is common in internal clastic structures, mainly spherical granular (Fig. 5a), fine-grained (Fig. 5b,c), granular (Fig. 5d), irregular block (Fig. 5e), and more terrigenous clastic material. Among them, the most common structure is the spherical granular structure. It has large particle sizes of approximately 200–500 μm and is ellipsoid, spherical, spindle, highly rounded and has an obvious boundary with dolomite. The fine-grained structure shows that the phosphate is filled with smaller particles of quartz or dolomite internally, which formed by absorbing the quartz and dolomite debris that formed in seawater during the phosphorite precipitation process. Some of the shells are wrapped around the dolomite and are approximately 10–20 μm thick, forming a granular structure. In addition, internal debris also appears in the irregular block structure. This shows that after phosphorite precipitation, the phosphorite was not damaged by strong seawater action or weaker seawater and was directly buried for mineralization.

However, the Zhijin phosphorite is common in bioclastic structures, such as conodonts (Fig. 5f), zones (Fig. 5g), and short columns (Fig. 5h), and a small amount of amorphous collophanite is also found (Fig. 5i). The conodont structure shows an open fan shape on one side and a closed cone on the other side, with particle sizes of 100–300 μm. Most conodont biological debris appears porous and relatively loose. The zonal structure shows ribbon-shaped biological debris, particle sizes of 10–1000 μm, and relatively large length and width ratios, mostly 8:1–10:1, with the characteristics of bending. Most zonal structures have rough surfaces, and some fragments have dense phosphorite inside. Short columnar structures are mainly short, cylindrical and partially dense. The particle sizes of columnar biological debris are 50–100 μm, and most of the debris are concave and uneven. The amorphous collophanite is in an unfixed form, cemented with dolomite, or distributed between dolomite crevices.

Mineralogical analysis indicated that during the Ediacaran period, there was no biological activity involved. After the deposition of phosphorite, the geochemical properties of pore water were stable, so the spherical phosphorite fragments of the Doushantuo Formation were preserved intact. During the early Cambrian period, algae and microorganisms proliferated and degraded after death. The released P formed phosphorite in pore water, resulting in the redistribution and differentiation of REEs and P. Therefore, some phosphorites in the Gezhongwu Formation exhibit destroyed biological residues, with amorphous collophanite and dolomite cemented together. The microorganisms were quickly buried after death, also leading to phosphorylation at the sedimentary interface.

### Chemical analysis

The 17 fresh samples were selected and crushed in a ceramic jaw crusher and powdered to ~ 200 mesh in an agate mill for whole-rock elemental and isotopic analyses. The whole-rock element analysis was carried out in the Australian Minerals Laboratory (Guangzhou) of the Aussie Analytical Testing Group.

Major element oxides were analyzed by the X-ray fluorescence (XRF) method (Rigaku RIX2100) using Li-borate glass disks. BCR-2 and GBW07105 were external standards, and the analytical accuracy and precision were both better than 5%.

Trace element concentrations were determined using an Agilent 7500a inductively coupled plasma mass spectrometry (ICP–MS) instrument. All samples were dissolved in a sealed high-temperature and high-pressure bomb using an equal proportion of superpure HF and HNO3. Details on the analytical procedure can be found in Liu et al.^22,23^ BHVO-2, AGV-2, BCR-2 and GSP-1 were used as standards, and the analytical accuracy was mostly better than 5% (relative error).

The REEs of all samples were measured via inductively coupled plasma atomic emission spectrometry (ICP‒AES) (Thermo iCAP-6300). The mineral composition and content were analyzed by FESEM‒EDS (FESEM; ZEISS Sigma-500, EDS; BRUKER XFlash 6|60) with an advanced mineral identification and characterization system (AMICS). The experimental conditions were as follows: the acceleration voltage was 20 kV, the resolution was 0.8 nm, and the probe current was 40–100 nA. The above analyses and testing were performed at the Hongchuang Testing Center of Nanjing University. δCe and δEu were calculated by δCe = Ce_N_/(La_N_ × Pr_N_)^1/2^ and δEu = Eu_N_/(Sm_N_ × Gd_N_)^1/2^, respectively, and N is the average of the post-Archean Australian shale standard value.^24^ All the original values for the major elements, trace elements and rare earth elements of the 17 samples are shown in Table 2.

**Table 2:** The original data of major elements (wt%), trace elements (ppm) by ICP-MS and rare earth elements (ppm) by ICP-AES in this study.

Samples	Zhijin														Weng’an		
	Dengying formation				Gezhongwu formation						Niutitang formation				Doushantuo formation		
	ZJ-1	ZJ-2	ZJ-3	ZJ-4	ZJ-5	ZJ-6	ZJ-7	ZJ-8	ZJ-9	ZJ-10	ZJ-11	ZJ-12	ZJ-13	ZJ-14	WA-1	WA-2	WA-3
SiO_2_	25.12	34.54	33.34	45.5	32.35	24.04	65.9	50.37	40.01	50.3	65.47	60.84	62.57	64.25	1.28	2.12	0.86
TiO_2_	0.01	0.01	0.02	0.04	0.06	0.07	0.05	0.40	0.06	0.07	0.46	0.79	0.81	0.62	0.01	0.09	0.02
Al_2_O_3_	0.36	0.38	0.22	0.80	1.40	1.99	0.34	6.47	0.68	0.27	18.16	17.02	19.20	15.31	0.22	0.49	0.26
BaO	0.03	0.01	0.03	0.03	0.06	0.09	0.26	0.08	0.05	0.06	0.05	0.13	0.09	0.59	0.06	0.10	0.07
CaO	41.30	31.60	38.40	27.60	33.00	32.00	2.80	12.60	30.70	24.30	0.20	1.34	0.02	0.75	52.90	53.10	52.80
TFe_2_O_3_	0.27	0.12	0.63	1.34	5.18	6.82	3.56	9.65	5.33	6.27	5.40	6.40	2.72	4.90	0.10	0.44	0.22
K_2_O	0.06	0.01	0.08	0.28	0.76	0.90	0.06	3.28	0.14	0.02	1.66	3.99	5.61	4.08	0.05	0.14	0.07
MgO	10.85	18.75	13.50	12.35	5.60	0.44	0.05	2.83	0.07	0.02	1.69	3.09	1.68	2.17	1.80	0.47	1.66
MnO	0.03	0.02	0.08	0.13	0.10	0.02	0.01	0.01	0.01	0.03	0.16	0.07	0.01	0.02	0.01	0.01	0.01
Na_2_O	0.24	0.05	0.05	0.03	0.08	0.12	0.03	0.02	0.08	0.07	1.16	1.07	0.08	0.04	0.25	0.37	0.41
P_2_O_5_	18.65	6.54	13.15	6.55	19.3	33.3	20.88	12.16	22.7	18.2	0.16	0.25	0.08	0.17	36.4	37.8	36.3
SO_3_	0.28	0.01	0.08	1.22	0.13	0.18	0.03	0.78	0.11	0.1	0.02	0.27	0.12	0.98	0.37	0.49	0.47
LOI 1000	24.32	45.41	31.53	28.05	17.07	13.02	10.52	18.61	11.39	10.92	3.09	4.83	6.65	11.60	5.76	3.05	5.70
V	13.0	6.0	18.0	13.0	33.0	82.0	130.0	188.0	192.0	22.0	66.0	181.0	668.0	782.0	6.0	14.0	9.0
Cr	10.0	10.0	10.0	10.0	10.0	30.0	60.0	100.0	90.0	10.0	10.0	42.0	140.0	120.0	10.0	30.0	10.0
Co	0.90	0.80	0.60	0.60	22.70	2.30	0.90	4.60	0.70	9.30	10.00	15.00	5.90	19.40	0.70	1.30	0.80
Ni	1.40	1.20	1.70	2.10	126.90	14.00	5.10	30.90	4.50	48.90	96.20	128.10	80.70	233.00	1.40	2.60	1.50
Cu	3.20	23.80	3.70	2.60	48.10	27.10	5.60	70.60	8.70	2.90	7.60	165.00	21.20	79.30	3.30	6.60	3.10
Zn	49	128	11	3	267	576	18	24	164	148	97	112	58	436	84	53	76
Rb	0.6	1.9	6.7	14.3	1.5	137.5	0.2	1.1	1.4	3.1	20.7	55.9	149.0	180.5	2.5	99.2	3.8
Sr	275	379	226	558	702	132.5	40.8	339	420	756	1100	45.2	78.1	44.9	55.6	153	355
Zr	3	2	8	12	15	90	3	6	8	4	42	196	192	159	7	42	3
Ba	790.5	579.5	770.5	979.5	1002	1863	2160	845	856	1041	1011	34	130	60	57	212	511
Hf	0.1	0.1	0.2	0.3	0.6	2.3	0.1	2.4	0.2	0.1	4	5.2	5.1	4.2	0.3	1.7	0.3
Th	7.19	13.40	15.00	11.80	3.52	2.00	0.52	1.71	1.60	0.50	0.29	0.05	0.54	0.15	3.31	3.39	3.34
U	9.88	20.06	35.80	23.80	21.15	15.60	5.18	10.25	9.90	2.90	4.77	0.62	2.88	0.18	4.05	3.66	4.26
Sc	0.60	1.50	0.10	2.50	1.60	5.10	7.20	9.60	15.30	18.70	0.80	7.40	6.00	5.00	0.60	0.20	0.70
La	8.60	2.40	124.50	74.50	197.00	337.00	47.40	85.60	168.50	109.50	21.10	40.60	9.80	39.30	9.20	36.50	45.30
Ce	10	10	10	10	10	30	40	100	90	10	50	110	60	120	10	30	10
Pr	2.45	2.25	20.50	11.10	36.80	58.20	12.65	23.20	30.60	21.30	5.75	9.42	2.64	7.99	1.59	7.97	6.58
Nd	13.70	1.30	95.20	51.70	182.00	275.00	56.70	103.50	136.50	94.20	28.90	36.40	12.50	29.40	8.00	38.60	30.00
Sm	3.46	0.25	17.10	9.16	34.10	51.70	13.50	23.10	26.60	16.95	8.56	7.04	3.01	4.87	1.48	8.06	6.03
Eu	0.98	0.09	4.14	2.09	11.00	11.85	3.99	5.54	7.34	4.14	1.69	1.21	0.65	0.76	0.32	1.53	1.30
Gd	4.38	0.29	19.90	11.25	38.20	57.20	15.25	23.10	34.40	19.65	7.90	6.14	3.17	4.02	1.59	9.46	8.95
Tb	0.66	0.04	2.83	1.60	4.98	8.11	2.35	3.32	4.89	2.78	1.17	1.00	0.45	0.56	0.22	1.24	1.23
Dy	3.95	0.21	17.20	9.92	28.20	48.40	13.40	17.95	31.10	15.95	6.48	5.95	2.44	3.48	1.26	6.52	7.01
Ho	0.83	0.05	3.68	2.22	5.66	9.83	2.82	3.48	6.82	3.49	1.22	1.25	0.47	0.72	0.28	1.25	1.41
Er	2.21	0.12	10.05	6.16	14.85	26.10	7.42	8.42	18.35	9.21	3.30	3.55	1.01	2.13	0.76	3.14	3.73
Tm	0.28	0.01	1.21	0.75	1.71	3.08	1.04	1.01	2.23	1.12	0.46	0.54	0.11	0.35	0.10	0.38	0.45
Yb	1.36	0.07	6.08	3.78	8.07	15.15	5.69	4.96	10.50	5.30	2.88	3.36	0.51	2.44	0.51	1.78	2.12
Lu	0.18	0.01	0.80	0.51	1.03	1.98	0.84	0.70	1.43	0.72	0.43	0.55	0.06	0.42	0.07	0.24	0.29
Y	36.7	10.9	204	127.5	308	476	285.2	131	332	259.5	36.7	34.4	17.4	19.8	8.8	40.6	48.9

*LOI* loss on ignition.

TFe_2_O_3_ = FeO/0.8998.

The Sr–Nd isotope separation test of rock samples was completed on the MC–ICP‒MS from the laboratory of Nanjing Hongchuang Detection Technology Co., Ltd. The Rb‒Sr and Sm‒Nd isotope separations of whole-rock samples included the processes of sample preparation, chemical separation, and testing (Table 3). The rock powder was dried in an oven and then weighed. Similar to the method of treating samples by ICP‒MS, the acid dissolution method was used to dissolve the sample. Rb‒Sr and Sm‒Nd isotope separation was carried out using the conventional cation exchange resin method. The type of resin used for Rb‒Sr separation was AG-SOW-X8, and HDEHP resin was used for Sm‒Nd separation. For detailed separation and test procedures, see Liu et al.^25^

**Table 3:** The Sr–Nd isotopic composition by MC-ICP-MS in this study.

Samples			Rb (ppm)	Sr (ppm)	^87^Rb/^86^Sr	^87^Sr/^86^Sr	2σ	(^87^Sr/^86^Sr)_i_	Sm (ppm)	Nd (ppm)	^147^Sm/^144^Nd	^143^Nd/^144^Nd	2σ	(^143^Nd/^144^Nd)_i_	εNd(t)	T_DM2_ (Ma)
Zhijin	Dengying formation	ZJ-1	0.6	275.0	0.01	0.710969	0.000005	0.710933	16.95	94.20	0.108718	0.511896	0.000007	0.511611	− 9.98	2729
		ZJ-2	1.9	379.0	0.01	0.709753	0.000005	0.709670	17.10	95.20	0.108528	0.511897	0.000007	0.511613	− 9.95	2725
		ZJ-3	6.7	226.0	0.09	0.709769	0.000005	0.709280	9.16	51.70	0.107051	0.511926	0.000008	0.511645	− 9.32	2648
		ZJ-4	14.3	558.0	0.07	0.709693	0.000005	0.709270	34.10	182.0	0.113205	0.511946	0.000007	0.511649	− 9.25	2640
	Gezhongwu formation	ZJ-5	1.5	702.0	0.01	0.710234	0.000004	0.710199	6.03	30.00	0.121445	0.512083	0.000008	0.511765	− 6.99	2367
		ZJ-6	137.5	132.5	3.00	0.710288	0.000005	0.710184	4.87	29.40	0.100084	0.512085	0.000009	0.511823	− 5.85	2228
		ZJ-7	0.2	40.8	0.01	0.710165	0.000010	0.710084	0.25	1.30	0.116193	0.511974	0.000013	0.511670	− 8.85	2591
		ZJ-8	1.1	339.0	0.01	0.709944	0.000005	0.709891	3.46	13.70	0.152595	0.512148	0.000007	0.511748	− 7.31	2405
		ZJ-9	1.4	420.0	0.01	0.710052	0.000005	0.709997	1.48	8.00	0.111778	0.512024	0.000013	0.511731	− 7.65	2446
		ZJ-10	3.1	756.0	0.01	0.710268	0.000005	0.710200	8.06	38.60	0.126163	0.512060	0.000008	0.511730	− 7.67	2449
	Niutitang formation	ZJ-11	20.7	1100	0.05	0.710403	0.000004	0.710093	51.70	275.0	0.113590	0.511952	0.000008	0.511655	− 9.14	2627
		ZJ-12	55.9	45.2	3.58	0.735188	0.000005	0.714804	8.56	28.90	0.178962	0.512144	0.000009	0.511675	− 8.75	2579
		ZJ-13	149.0	78.1	5.52	0.746877	0.000004	0.715432	7.04	36.40	0.116857	0.512034	0.000009	0.511728	− 7.71	2453
		ZJ-14	180.5	44.9	11.63	0.786746	0.000007	0.720486	13.50	56.70	0.143858	0.512081	0.000009	0.511705	− 8.16	2509
Weng’an	Doushantuo formation	WA-1	2.5	55.6	0.13	0.712145	0.000007	0.711404	3.01	12.50	0.145492	0.511910	0.000008	0.511529	− 11.60	2923
		WA-2	99.2	153.0	1.88	0.720956	0.000006	0.710269	23.10	103.50	0.134851	0.511892	0.000011	0.511539	− 11.40	2899
		WA-3	3.8	355.0	0.03	0.710803	0.000005	0.710627	26.60	136.50	0.117742	0.511906	0.000008	0.511597	− 10.26	2762

ε_Nd_(t) = 1/λ × ln[1 + [(^143^Nd/^144^Nd)_DM_-(^143^Nd/^144^Nd)_S_]/[(^147^Sm/^144^Nd) − (^147^Sm/^144^Nd)_S_]].

Two-stage Nd model ages (TDM2) were calculated by adopting ^147^Sm/^144^Nd of 0.118 for average continental crust^51^, using the formula of Liew and Hofmann^52^. Subscripts: *m* measured, *DM* depleted mantle. As for CHUR: ^147^Sm/^144^Nd = 0.1967^53^, ^143^Nd/^144^Nd = 0.0336^53^; λ^147^Sm = 6.54E−12 yr^−1^^54^; λ^87^Rb = 1.42E−11 yr^−1^^55^; Subscripts: *m* measured, *CHUR* chondritic reservoir.

## Results

The statistics of the major elements, trace elements, rare earth elements and different element ratios are calculated and listed in Table 4.

**Table 4:** The statistics of the major elements, trace elements and rare earth elements compositions in this study.

Samples	Zhijin phosphorite deposit									Weng’an phosphorite deposit		
	Dengying formation			Gezhongwu formation			Niutitang formation			Doushantuo formation		
Elements and ratios	Min	Max	Mean	Min	Max	Mean	Min	Max	Mean	Min	Max	Mean
SiO_2_	25.12	45.50	35.31	24.04	65.90	44.97	60.84	65.47	63.16	0.86	2.12	1.49
TiO_2_	0.01	0.04	0.02	0.05	0.40	0.12	0.46	0.81	0.67	0.01	0.09	0.04
Al_2_O_3_	0.22	0.80	0.44	0.27	6.47	1.86	15.31	19.20	17.42	0.22	0.49	0.32
BaO	0.01	0.03	0.03	0.05	0.26	0.10	0.05	0.59	0.22	0.06	0.10	0.08
CaO	27.60	41.30	34.73	2.80	33.00	22.57	0.02	1.34	0.58	52.80	53.10	52.93
TFe_2_O_3_	0.12	1.34	0.59	3.56	9.65	6.14	2.72	6.40	4.86	0.10	0.44	0.25
K_2_O	0.01	0.28	0.11	0.02	3.28	0.86	1.66	5.61	3.84	0.05	0.14	0.09
MgO	10.85	18.75	13.86	0.02	5.60	1.50	1.68	3.09	2.16	0.47	1.80	1.31
MnO	0.02	0.13	0.07	0.01	0.10	0.03	0.01	0.16	0.07	0.01	0.01	0.01
Na_2_O	0.03	0.24	0.09	0.02	0.12	0.07	0.04	1.16	0.59	0.25	0.41	0.34
P_2_O_5_	0.06	18.65	9.60	18.20	33.30	25.75	0.08	0.25	0.17	36.30	37.80	36.83
SO_3_	0.01	1.22	0.40	0.03	17.80	3.06	0.02	0.98	0.35	0.37	0.49	0.44
LOI 1000	24.32	45.41	32.33	10.52	18.61	13.59	3.09	11.60	6.54	3.05	5.?6	4.84
Fe/Ti	14.00	39.08	30.33	28.15	113.67	88.96	3.92	13.70	9.07	5.70	12.83	10.07
(Fe + Mn)/Ti	16.57	43.25	34.26	28.18	114.03	89.55	3.93	14.14	9.23	5.85	13.48	10.76
Al/(Al + Fe + Mn)	0.19	0.67	0.41	0.03	0.34	0.15	0.67	0.84	0.73	0.45	0.80	0.50
K_2_O + Na_2_O	0.06	0.31	0.20	0.09	3.30	0.93	2.82	5.B9	4.42	0.30	0.51	0.43
SiO_2/_Al_2_O_3_	56.88	151.55	104.26	7.79	186.3	97.05	3.26	4.20	3.74	3.31	5.82	4.57
CaO/(Fe + CaO)	0.97	1.00	0.99	0.53	0.90	0.80	0.01	0.23	0.12	0.99	1.00	1.00
(MgO/Al_2_O_3_) × 100	1543.75	6136.36	3907.05	7.41	400.00	83.04	8.75	18.16	12.60	95.92	818.18	517.52
V	6.00	18.00	12.50	22.00	192.00	107.83	66.00	782.00	424.25	6.00	14.00	9.67
Cr	10.00	10.00	10.00	10.00	100.00	50.00	10.00	140.00	78.00	10.00	30.00	16.67
Co	0.80	0.90	0.73	0.70	22.70	6.75	5.90	19.40	12.58	0.70	1.30	0.93
Ni	1.20	2.10	1.60	4.50	126.90	38.38	80.70	233.00	134.50	1.40	2.60	1.83
Cu	2.60	23.80	8.33	2.90	70.60	27.17	7.60	165.00	68.28	3.10	6.60	4.33
Zn	3.00	128.00	47.75	18.00	576.00	199.50	58.00	436.00	175.75	53.00	84.00	71.00
Rb	0.80	14.30	5.88	0.20	137.50	24.13	20.70	180.50	101.53	2.50	99.20	35.17
Sr	226.00	558.00	359.50	40.80	756.00	398.38	44.90	1100.00	317.05	55.60	355.00	187.87
Zr	2.00	12.00	6.25	3.00	90.00	21.00	42.00	196.00	147.25	3.00	42.00	17.33
Ba	579.50	979.50	780.00	845.00	2160.00	1294.50	34.00	1011.00	308.75	57.00	511.00	260.00
Hf	0.10	0.30	0.18	0.10	2.40	0.95	4.00	5.20	4.63	0.30	1.70	0.77
Th	7.19	15.00	11.85	0.50	3.52	1.64	0.05	0.54	0.26	3.31	3.39	3.35
U	9.88	35.80	22.39	2.90	21.15	10.83	0.18	4.77	2.11	3.66	4.26	3.99
Sc	0.10	2.50	1.18	1.60	18.70	9.58	0.80	7.40	4.80	0.20	0.70	0.50
Ba/Sr	1.53	3.41	2.39	1.38	52.94	12.39	0.75	l.66	1.17	1.03	2.04	1.50
U/Th	1.37	2.39	1.82	5.80	9.96	6.96	1.20	16.45	8.85	1.07	1.27	1.19
Ni/Co	1.50	3.50	2.35	5.26	6.72	5.96	8.54	13.68	10.96	1.88	2.00	1.96
V/Cr	0.60	1.80	1.25	1.88	3.30	2.40	4.31	6.60	5.55	0.47	0.90	0.66
V/Sc	4.00	180.00	52.72	1.18	20.63	14.68	24.46	156.40	93.67	10.00	70.00	30.95
V/(V + Ni)	0.83	0.91	0.88	0.21	0.98	0.70	0.41	0.89	0.67	0.81	0.86	0.84
Zr/Rb	0.84	10.00	3.27	0.65	15.00	6.35	0.88	3.51	1.93	0.42	2.80	1.34
Co/Zn	0.01	0.20	0.10	0.00	0.19	0.10	0.04	0.13	0.09	0.01	0.02	0.15
La	2.40	124.50	52.50	47.40	337.00	157.50	9.80	40.60	27.70	9.20	45.30	30.33
Ce	10.00	10.00	10.00	10.00	100.00	46.67	50.00	120.00	85.00	10.00	30.00	16.67
Pr	2.25	20.50	9.08	12.65	58.20	30.46	2.64	9.42	6.45	1.59	7.97	5.38
Nd	1.30	95.20	40.48	56.70	275.00	141.32	12.50	36.40	26.80	8.00	38.80	25.53
Sm	0.25	17.10	7.49	13.50	51.70	27.66	3.01	8.56	5.87	1.48	8.08	5.19
Eu	0.09	4.14	1.83	3.99	11.85	7.31	0.65	1.69	1.08	0.32	1.53	1.05
Gd	0.29	19.90	8.96	15.25	57.20	31.30	3.17	7.90	5.31	1.59	9.46	6.67
Tb	0.04	2.83	1.28	2.35	8.11	4.41	0.45	1.17	0.80	0.22	1.24	0.90
Dy	0.21	17.20	7.82	13.40	48.40	25.83	2.44	6.48	4.59	1.26	7.01	4.93
Ho	0.05	3.68	1.70	2.82	9.83	5.35	0.47	1.25	0.92	0.28	1.41	0.98
Er	0.12	10.05	4.64	7.42	26.10	14.06	1.01	3.55	2.50	0.76	3.73	2.54
Tm	0.01	1.21	0.56	1.01	3.08	1.70	0.11	0.54	0.37	0.10	0.45	0.31
Yb	0.07	6.08	2.82	4.96	15.15	8.28	0.51	3.36	2.30	0.51	2.12	1.47
Lu	0.01	0.80	0.38	0.70	1.98	1.12	0.06	0.55	0.37	0.07	0.29	0.20
Y	10.90	204.00	9478	131.00	476.00	298.62	17.40	36.70	27.08	8.80	48.90	32.77
REE	27.99	537.19	244.29	508.25	1409.60	801.57	114.22	261.41	197.10	44.18	187.27	134.92
LREE	16.29	271.44	121.37	174.24	763.75	410.91	88.60	204.67	152.90	30.59	122.66	84.15
HREE	11.70	265.75	122.92	193.94	645.85	390.66	25.62	60.54	44.21	13.59	74.09	50.76
LREE/HREE	0.78	1.39	1.04	0.52	1.76	1.08	1.92	5.96	3.74	1.34	2.25	1.83
δCe	0.05	0.99	0.41	0.03	0.52	0.22	1.05	1.62	1.38	0.13	0.80	0.38
δEu	0.97	1.57	1.20	1.03	1.44	1.19	0.81	0.99	0.91	0.83	0.98	0.88
δY	1.48	2.17	1.83	1.32	3.70	2.22	1.00	1.30	1.09	1.13	1.24	118
δPr	1.23	3.91	2.92	1.34	5.07	2.91	0.57	0.89	0.78	1.05	2.23	1.55

### Characteristics of major elements

The contents of major elements differ in rocks from different strata of the Zhjin and Weng’an deposits. Generally, the SiO_2_ content in dolomites from the Dengying Formation of the Zhijin deposit is low, with an average value of 35.31% and values ranging from 25.12 to 45.50%. The MgO and CaO contents are high, with average values of 13.86% and 34.73%, ranging from 10.85 to 18.75% and 27.60 to 41.30%, respectively. Al_2_O_3_, TiO_2_ and (K_2_O + Na_2_O) contents are very low. TFe_2_O_3_ values vary in the range of 0.12–1.34%, with an average value of 0.59%. In addition, the LOI values in samples from the dolomites of the Dengying Formation are very high, with an average value of 32.33% and values varying from 24.32 to 45.41.

The phosphorites from the Gezhongwu Formation contain very high SiO_2_, with an average value of 44.97% and values varying in a wide range from 24.04 to very 65.90%. These phosphorites contain high TFe_2_O_3_, with an average value of 6.14%. The CaO content is intermediate in comparison with rocks from other formations, with an average value of 20.68%. However, MgO (average value of 1.50%), Al_2_O_3_, TiO_2_ and (K_2_O + Na_2_O) are low. A relatively high LOI is observed in phosphorites from the Gezhongwu Formation, with an average value of 13.59% and values ranging from 10.52 to 18.61%.

The carbonaceous shale from the Niutitang Formation of the Zhijin deposit is characterized by high contents of SiO_2_ and Al_2_O_3_, with average values of 63.16% and 17.42%, respectively, with values varying from 60.84.25 to 65.47% and 15.31 to 19.20%, respectively. TFe_2_O_3_ is higher, with an average value of 4.86% and values ranging from 2.72 to 6.40%. Obvious scattered spots of pyrite are observed on the rock surface. However, there are low contents of CaO and MgO in these shales, with average values of 0.58% and 2.16%, respectively; however, the (K_2_O + Na_2_O) content is high, with an average value of 4.42%.

It is noteworthy that the major element contents in the Doushantuo Formation vary greatly from those in the Gezhongwu Formation of the Zhijin deposit. The phosphorite from the Doushantuo Formation of the Weng’an deposit is characterized by a high CaO content (the average value is 52.93%), low SiO_2_ content (average value of 1.49%), Al_2_O_3_ content of 0.32%, TFe_2_O_3_ content of 0.25% and (K_2_O + Na_2_O) content of 0.43%. Moreover, the content of MgO is low, with an average value of 1.31%. However, the content of P_2_O_5_ (the average value is 36.83%) in the Doushantuo Formation of the Weng’an deposit (the average value is 16.08%) is much higher than that from the Gezhongwu Formation of the Zhijin deposit.

### Characteristics of trace elements

**Figure 6:**
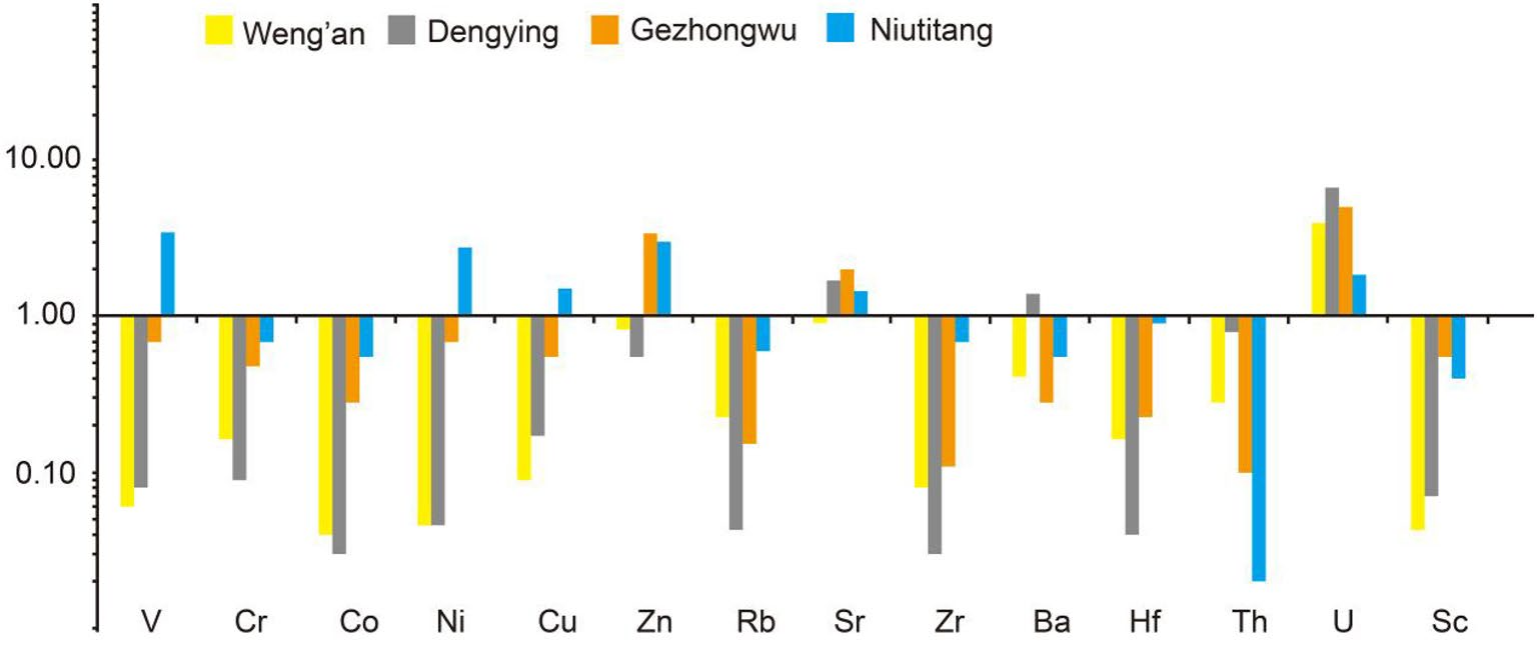
PAAS-normalized trace element distribution patterns of samples from the Zhijin and Weng’an phosphorite deposits (The data correspond to the mean value of samples from each formation in Table 1).

The PAAS normalization of trace elements (PAAS data are from Taylor and Mclennan^26^) for the samples collected in this study indicated that the enrichment degree of trace elements differs in different strata from the Zhijin and Weng’an phosphorite deposits. Among them, the dolomite from the Dengying Formation is rich in Sr, Ba and U; phosphorites from the Gezongwu Formation of the Zhijin deposit are characterized by enrichment in Zn, Sr, and U; and carbonaceous shales from the Niutitang Formation are enriched in V, Ni, Zn, Sr, and U, as shown in Fig. 6. The enrichment degrees of Sr and Ba vary in different strata in the two deposits. Moreover, the enrichment degrees of different trace elements in the Gezhongwu Formation vary partially.

### Geochemical characteristics of rare earth elements

**Figure 7:**
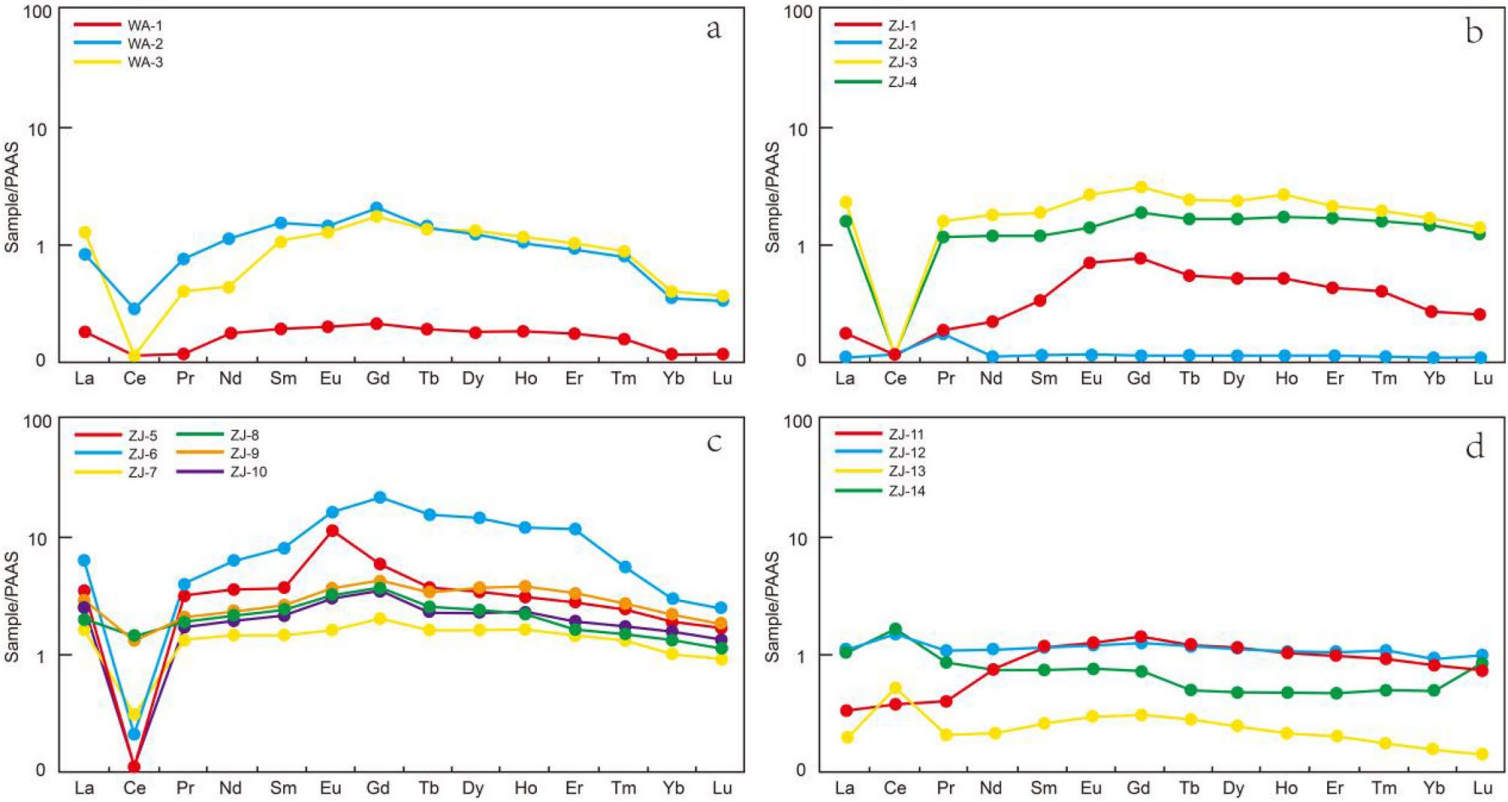
PAAS-normalized rare earth element distribution patterns of samples from the Zhijin and Weng’an phosphorite deposits. (**a**) Phosphorites from the Doushantuo Formation. (**b**) Dolomite from the Dengying Formation. (**c**) Phosphorites from the Gezhongwu Formation. d Carbonaceous shale from the Niutitang Formation.

REEs in phosphorite deposits from the Zhijin and Weng’an deposits in Guizhou vary greatly in different strata (Fig. 7).

The Gezhongwu Formation contains the highest REE content in this study, with an average ΣREE content of 801.57 ppm and values varying from 508.25 to 1409.6 ppm. LREE/HREE ratios vary from 0.81 to 2.05, with an average of 1.33. The PAAS normalization pattern is left-leaning, with cap-type distribution characteristics and a significant negative Ce anomaly (varying from 0.03 to 0.52, with an average value of 0.22). δEu values vary from 1.03 to 1.44, with ZJ-5 exhibiting a high δEu of 1.44. Most samples have δEu values of approximately 1, exhibiting a positive Eu anomaly.

Dolomite from the Dengying Formation contains highly variable ΣREEs from 5.99 to 527.19 ppm, with an average value of 236.29 ppm. The LREE/HREE ratios range from 0.78 to 4.92, and the average value is 1.92, exhibiting enrichment in LREEs. The samples have negative Ce anomalies (varying from 0.05 to 0.99, with an average of 0.41); in addition, most samples exhibit positive Eu anomalies, except sample ZJ-4.

The carbonaceous shale from the Niutitang Formation contains relatively low ΣREEs, varying from 114.22 to 259.61 ppm, with an average content of 196.65 ppm. LREE/HREE ratios show high fractionation between LREEs and HREEs, varying from 1.92 to 5.96, with an average of 3.74, indicating distinct LREE enrichment. It is noteworthy that the shale shows significant positive Ce anomalies and slight negative Eu anomalies.

Comparatively, the samples from the Gezhongwu Formation of the Weng’an deposit contain very low ΣREE contents varying from 44.38 to 177.30 ppm, with an average value of 136.32 ppm. The LREE/HREE ratios range from 1.34 to 2.25, with an average ratio of 1.83, indicating slight enrichment of LREEs.

Most samples in this study exhibit similar PAAS normalization patterns, except for samples from the carbonaceous shale of the Niutitang Formation.

### Sr–Nd isotopic composition

The values of the whole-rock Sr–Nd isotope test data for the samples are shown in Table 3. The results of the analysis showed that the (^87^Sr/^86^Sr)_i_ values of the Doushantuo Formation range from 0.709997 to 0.710200, and the ε_Nd_(t) values range from -7.67 to -6.99. The (^87^Sr/^86^Sr)_i_ values of the Dengying Formation range from 0.709944 to 0.710165, and the ε_Nd_(t) values range from − 8.85 to − 7.31. The (^87^Sr/^86^Sr)_i_ values of the Gezhongwu Formation range from 0.709693 to 0.720956, and the ε_Nd_(t) values range from − 11.60 to − 9.14. The (^87^Sr/^86^Sr)_i_ values of the Niutitang Formation range from 0.714804 to 0.720486, and the ε_Nd_(t) values range from − 5.85 to − 8.7.

It is generally believed that the high ε_Nd_(t) value and low two-stage pattern age (TDM2) value indicate that the source rock may originate from nascent crust in circulation or with nascent mantle material added to the continental crust. The low ε_Nd_(t) value and high TDM2 value indicate that the source rock may have originated from deep crustal material or the remelting of ancient crustal material^27^. The Doushantuo Formation has high TDM2 values varying from 2761 to 2923 Ma, with an average value of 2861 Ma, low ε_Nd_(t) values varying from − 10.26 to − 11.60, with an average value of − 11.09, and relatively high initial Sr values varying from 55.6 to 355.0, with an average value of 187.9.

**Figure 8:**
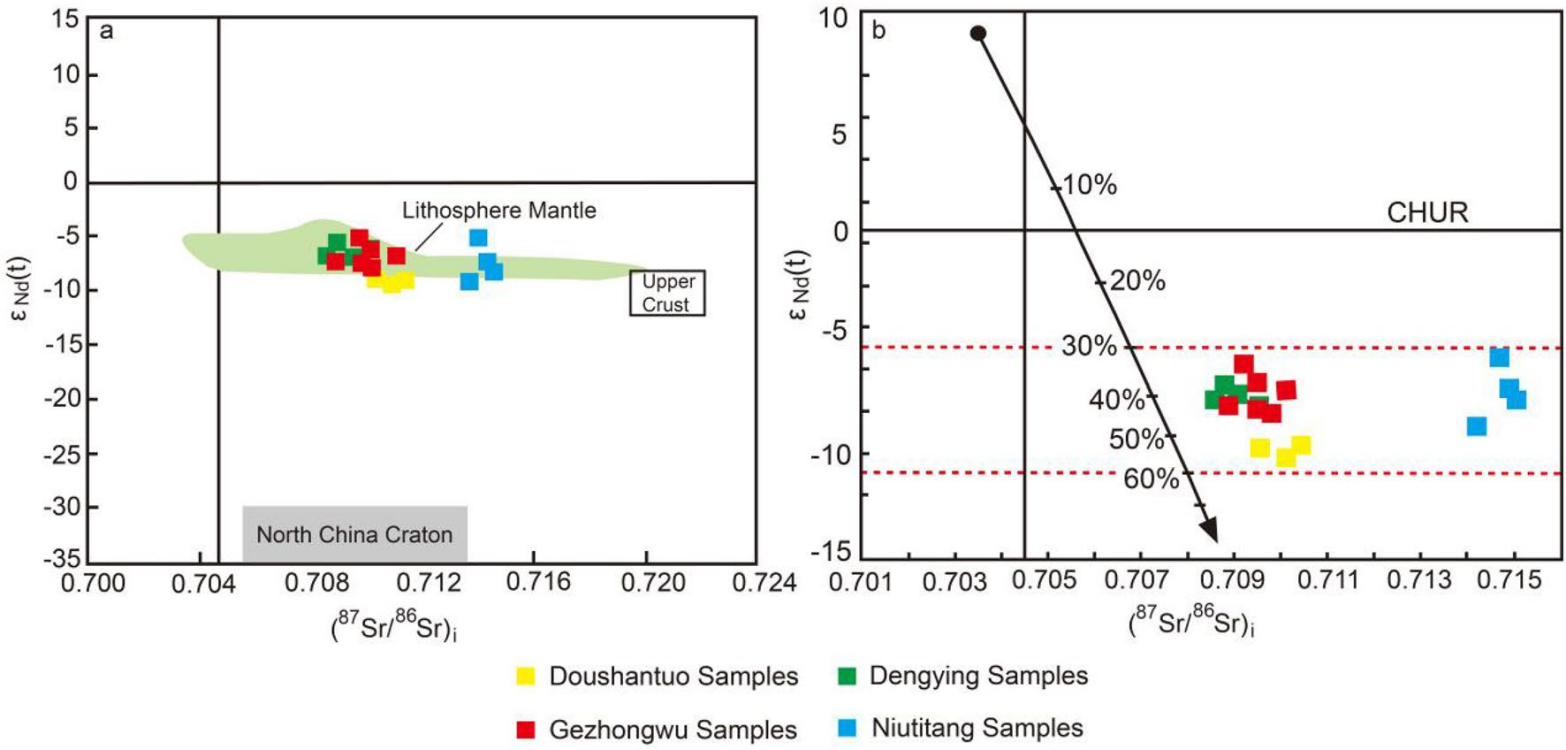
ε_Nd_(t) − (^87^Sr/^86^Sr)_i_ diagram of samples from the Zhijin and Weng’an phosphorite deposits. The upper crust data are from Jahn et al.^28^. The ancient lithospheric mantle data are from Ma et al.^29^.

In Fig. 8a, the samples plot between the upper crust and the mantle area, showing the characteristics of mantle mixing. The average TDM2 value of the Doushantuo Formation is 2861 Ma, while the average ε_Nd_(t) value (− 11.60 to − 10.26) is − 11.09, reflecting that the rock in the source region is associated with partial melting of Neoarchean crustal material. The average TDM2 value (2228–2729) of the Gezhongwu Formation is 2414 Ma, while the average ε_Nd_(t) value (− 8.85 to − 5.85) is − 7.39, which is higher than that of the samples in the Doushantuo Formation. In Fig. 8a, the samples plot in the ancient lithospheric mantle region, indicating that the source rock may have formed in the process of ancient crustal reconstruction caused by volcanic activity. The average ε_Nd_(t) values of the Dengying Formation and Niuhootang Formation are − 9.62 and − 8.44, respectively, and the average TDM2 values are 2686 Ma and 2542 Ma, respectively, reflecting that the Dengying Formation contains more ancient crustal material in the source rock.

In addition, in Fig. 8b, a simple simulation calculation of Sr–Nd isotopes indicates that the contribution from ancient crustal material is approximately 30–60%, which is within the range of the red dotted line. The order of the contribution of ancient crust material is the Doushantuo Formation > Dengying Formation > Niutitang Formation > Gezhongwu Formation. Among them, the contribution of the ancient crustal material in the Doushantuo Formation is the highest, accounting for more than 50%.

## Discussion

### Material sources analysis

In this study, it is indicated that although the grade of phosphorite from the Doushantuo Formation of the Weng’an deposit is much higher than that from the Gezhongwu Formation of the Zhijin deposit, the REE content in the latter is much higher than that in the former. It is then deduced that the supply of REEs is the key factor controlling the REE content in phosphorite. The identification of REE material sources is thus very important to understand the enrichment mechanism of REEs in sedimentary phosphorite.

Usually, marine deposition is composed of extrinsic material, including terrigenous material carried by river runoff, aeolian or glacial debris, and endogenous materials, including volcanogenic material and related alteration products and hydrothermal materials. In addition, precipitation from seawater is another important material provider. To date, most studies have focused more on the material sources of phosphorus in phosphorite from Southwest China with good ore quality, few studies have been performed on the REE material source, and no consensus has been reached for the REEs enriched in phosphorite from the Gezhongwu Formation. Possible REE material sources include hydrothermal material and related alteration products^6^. Alternatively, at appropriate temperatures, REEs and phosphorite exchange substances in pore water, resulting in enrichment of REEs in phosphorite^30^. Wu et al. believed that the enrichment of REEs may be affected by later diagenetic weathering. The sources of REEs are diverse, and various factors need to be studied in more detail^1^.

However, concerning the uneven distribution of REEs from phosphorites from the Gezhongwu Formation and Doushantuo Formation, it is deduced to be impossible for local seawater to be a main REE supplier with great variation in chemical composition in the two periods. In addition, previous studies reveal that the Kunyang phosphorite, which formed in the same geological period as the Zhijin phosphorite, contains very low REE contents^9^. In this study, to identify the exact REE suppliers for the Zhijin phosphorite, we used the geochemical index to sort out the possible material sources with analysis of the depositional environment and geological tectonic evolution.

#### Evidence from major elements

The SiO_2_/Al_2_O_3_ ratio has been widely used to distinguish different material sources for rocks since it was first suggested by Taylor & Mclennan^26^. The SiO_2_/Al_2_O_3_ ratio in this study varies greatly (Table 4) in the sequence of phosphorite from dolomite from the Dengying Formation (104.22) > Gezhongwu Formation (97.05) > phosphorite from Doushantuo (4.57) > carbonaceous shale from the Niutitang Formation (3.73). Thus, phosphorite from the Dengying and Gezhongwu Formations of the Zhijin deposit is obviously impacted by hydrothermal activity.

**Figure 9:**
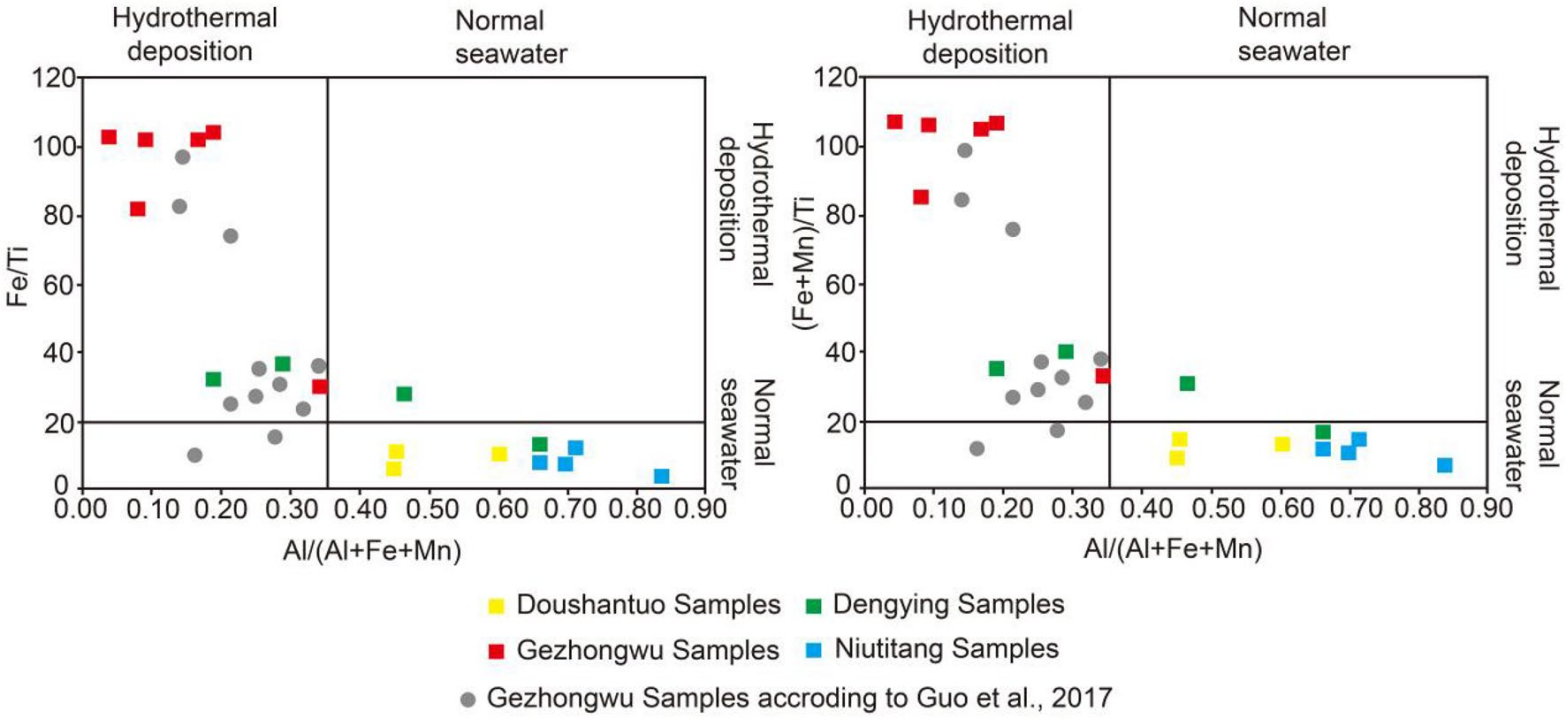
The ratios of Fe/Ti, (Fe + Mn)/Ti and Al/(Al + Fe + Mn) of samples from the Zhijin and Weng’an phosphorite deposits.

Fe, Mn, Al and Ti are further used to carry out the source study. It is generally believed that the Al-rich marine sediments are related to the intervention of terrigenous materials, while the enrichment of Fe and Mn is mainly affected by the participation of hydrothermal fluid. Therefore, Al/(Al + Fe + Mn) is often used to evaluate the hydrothermal influence^31^. In this sense, marine sediments related to terrigenous material show Fe/Ti > 20, (Fe + Mn)/Ti > 25, and Al/(Al + Fe + Mn) > 0.5, and the source of hydrothermal action is indicated by Al/(Al + Fe + Mn) < 0.35^31^. Therefore, Fe/Ti–Al/(Al + Fe + Mn) and (Fe + Mn)/Ti–Al/(Al + Fe + Mn) are used to distinguish the rock provenance in this study. The results show that all samples of the Gezhongwu Formation and two of the samples of the Dengying Formation dolomite plot in the hydrothermal sedimentary area (Fig. 9), which is consistent with previous research conclusions^6^.

#### Evidence from trace elements

**Figure 10:**
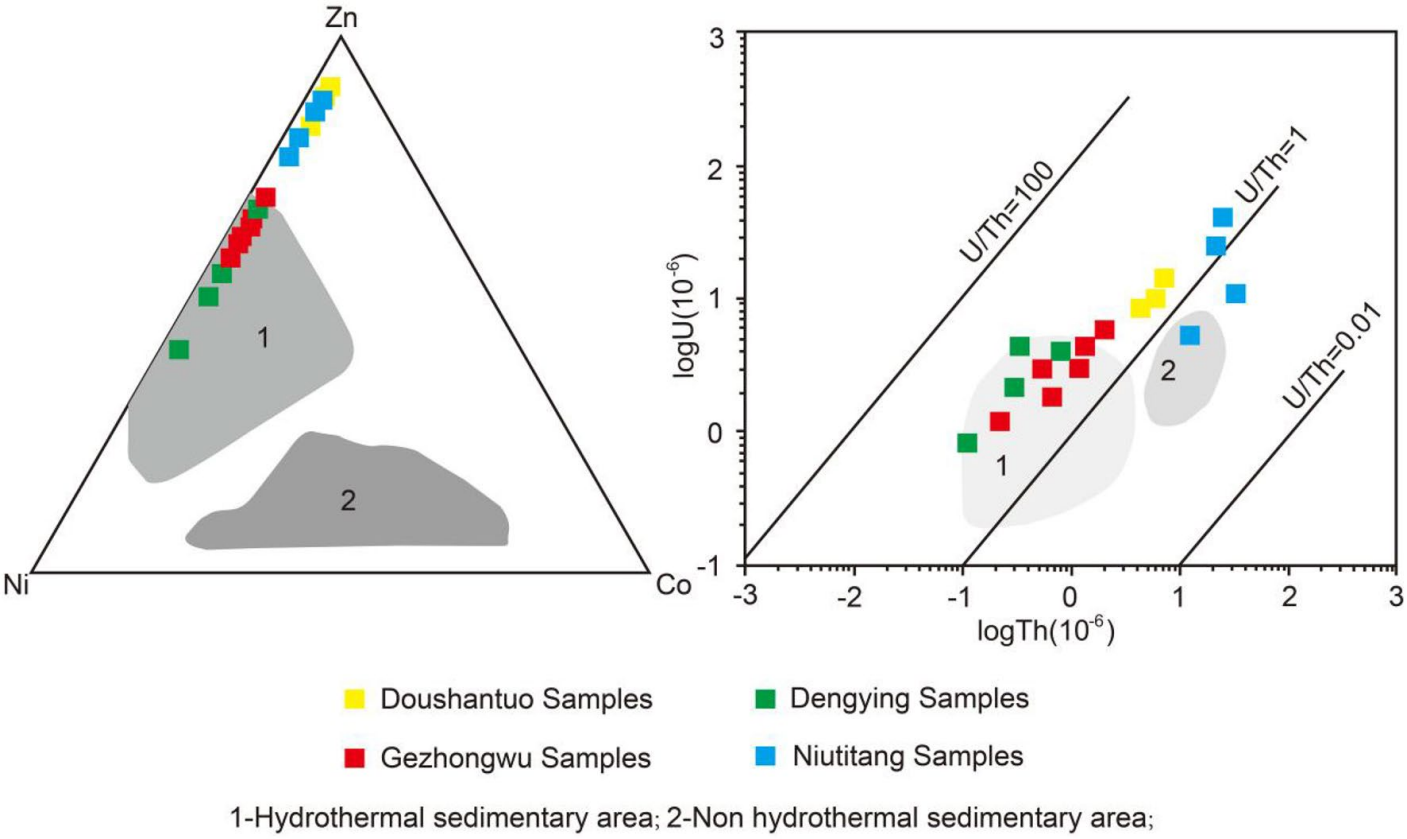
Co–Ni–Zn and U–Th diagram of samples from the Zhijin and Weng’an phosphorite deposits.

In general, it is believed that Cu, Ni and Zn are primary hydrothermal sources that are also involved in biological productivity^32^. The Co/Zn ratio of hydrothermal-sourced sediments is relatively low, with an average ratio of 0.15, while it is much higher in ferromanganese crusts, at approximately 2.5^33^. In this study, Co/Zn in all samples is very low. The Co/Zn ratios in the Weng’an samples range from 0.01 to 0.02, while the Co/Zn ratios in the Zhijin samples range from 0.01 to 0.20. With the usage of the Zn–Ni–Co ternary diagram (Fig. 10), it is noteworthy that the phosphorite from the Gezhongwu Formation and dolomite from the Dengying Formation are located in the hydrothermal sedimentation area and vicinity, showing distinct involvement of hydrothermal sedimentation.

The U/Th ratio can also be a proxy of redox conditions in paleo-seawater^34^. The U/Th ratio further confirms the impacts of hydrothermal activities, especially for phosphorite from the Gezhongwu Formation and dolomite from the Dengying Formation. Usually, normal sedimentary rock has U/Th < 1, while hydrothermal sedimentary rock has U/Th > 1^35^. The U/Th ratio (as shown in Table 4) in this study increases in the following sequence: carbonaceous shale from the Niutitang Formation (average value of 0.99, with values varying from 0.26 to 2.02) → phosphorite from the Doushantuo Formation (average value of 2.53, with values varying from 2.38 to 2.77) → dolomite from the Dengying Formation (12.26, with values varying from 5.33 to 16.45) → phosphorite from the Gezhongwu Formation (average of 18.23, with values varying from 5.99 to 56.81), showing the increasing impact from hydrothermal activities. The relationship between U and Th (Fig. 10) shows that the phosphorite from the Gezhongwu Formation and dolomite from the Dengying Formation are located in an area indicating paleohydrothermal sedimentation.

#### Evidence from rare earth elements

**Figure 11:**
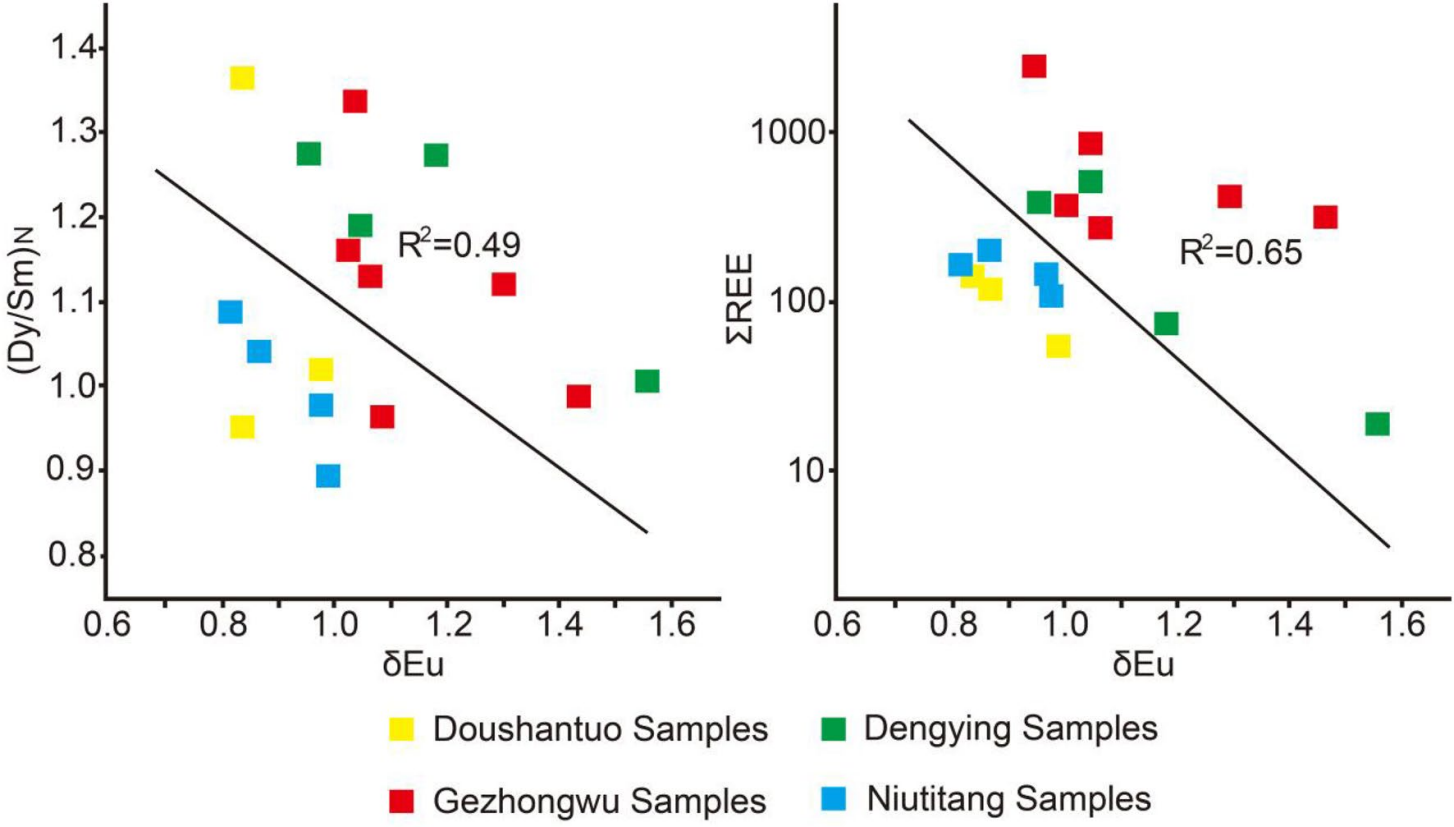
The (Dy/Sm)N-δEu and REE-δEu diagrams of samples from the Zhijin and Weng’an phosphorite deposits.

Generally, positive Eu anomalies are widely used to indicate the involvement of hydrothermal materials. In this study, δEu values vary from high to low in the sequence of from the Dengying Formation dolomite (1.20, 0.97–1.57) > Gezhongwu Formation phosphorite (1.19, 1.03–1.44) > Niutitang Formation carbonaceous shale (0.91, 0.81–0.99) > Doushantuo Formation phosphorite (0.88, 0.83–0.98). Particularly, with the increase in the REE content in the bulk sediment of phosphorite from the Gezhongwu Formation, the enrichment of REEs is found to be closely related to the negative Eu anomaly (Fig. 11). Moreover, phosphorite from the Doushantuo Formation of the Weng’an deposit exhibits obvious negative Eu anomalies, indicating that REE-rich phosphorite from the Gezhongwu Formation developed with the involvement of hydrothermal fluid. The impact of hydrothermal activity continued until the deposition of the Dengying Formation.

**Figure 12:**
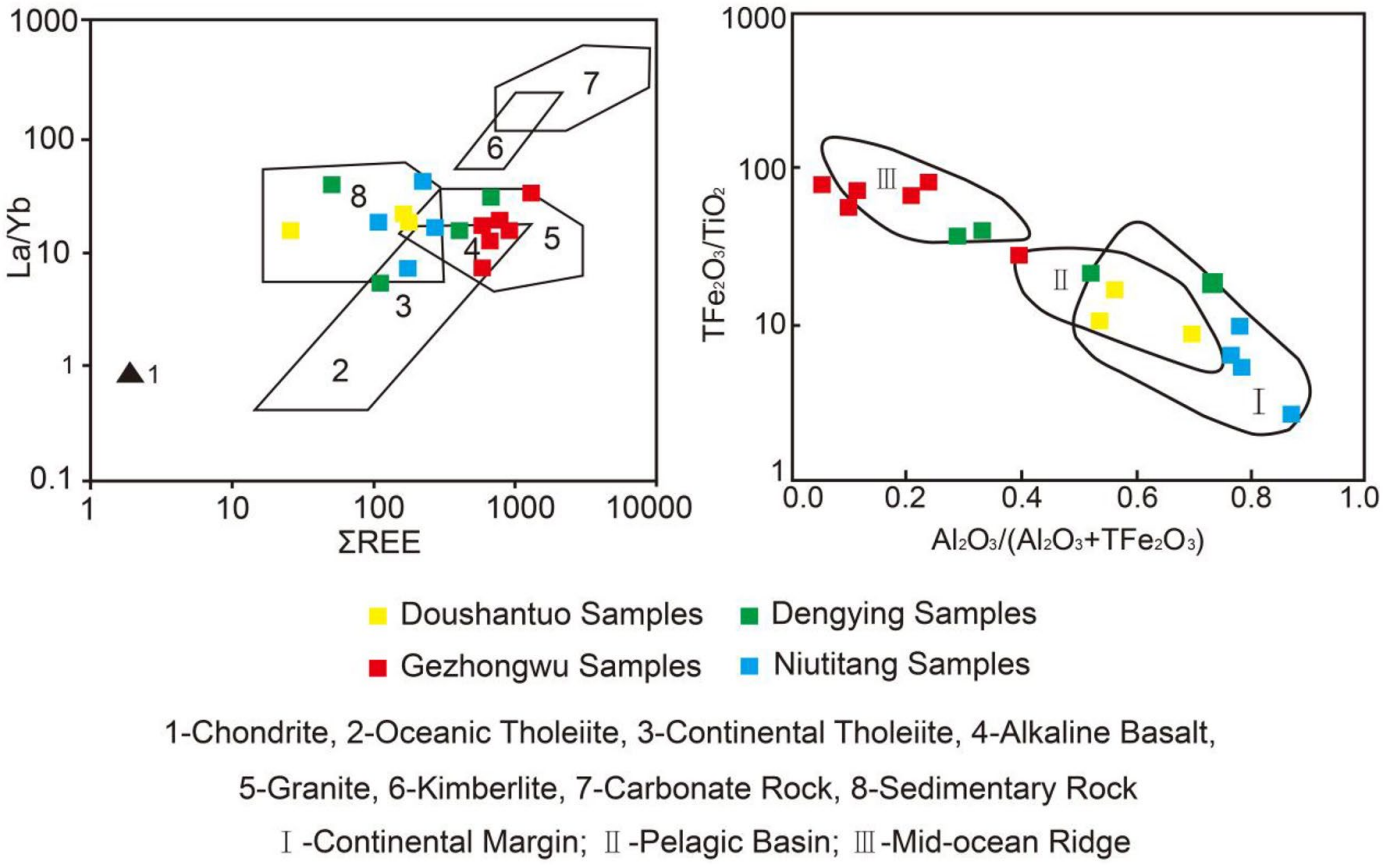
The La/Yb-REE and tectonic discrimination diagram.

A positive Y anomaly is also a geochemical indicator of hydrothermal sedimentation^36^. The average values of δY in this study vary from high to low from the Gezhongwu Formation phosphorite (2.22) > Dengying Formation dolomite (1.83) > Doushantuo Formation phosphorite (1.18) > Niutitang Formation carbonaceous shale (1.08); this sequence reflects that the phosphorite from the Gezhongwu Formation was formed with strong hydrothermal participation. Its highest REE content is then deduced to be closely related to hydrothermal activity. In the La/Yb REE plot, Fig. 12 shows that most phosphorite from the Gezhongwu Formation is located in the intersection area of submarine alkaline basalt and granite, which further suggests the participation of hydrothermal fluid from deep sources during the process of phosphorite sedimentation.

### Deposition environment analysis

The Mn/TiO_2_ ratio has been proven to be useful and widely accepted in identifying depositional environments^37^. A value ranging from 0.5 to 3.5 indicates that the deep sea or seabed is far from the mainland; a value less than 0.5 indicates a shallow sea or continental slope. The MnO/TiO_2_ ratios of the samples in this study are all within the range of 0.5–3.5, indicating a shallow marine sedimentary environment. In addition, Murray^38^ proposed a diagram of Al_2_O_3_, TFe_2_O_3_, and TiO_2_, and the La/Y ratio is adopted in this study to distinguish the tectonic environment (Fig. 12). All samples from the Niutitang Formation were collected from the continental margin area, and all samples from the Doushantuo Formation were collected from the overlapping area of the continental margin and pelagic basin. However, it is noteworthy that most samples from the Gezhongwu Formation were collected from the area of the mid-ocean ridge or the vicinity together with two samples from the Dengying Formation. Thus, both the Zhijin and Weng’an phosphorite deposits developed in bathyal or abyssal environments near continents. However, samples from the Dengying and Gezhongwu Formations are closely related to hydrothermal activity.

**Figure 13:**
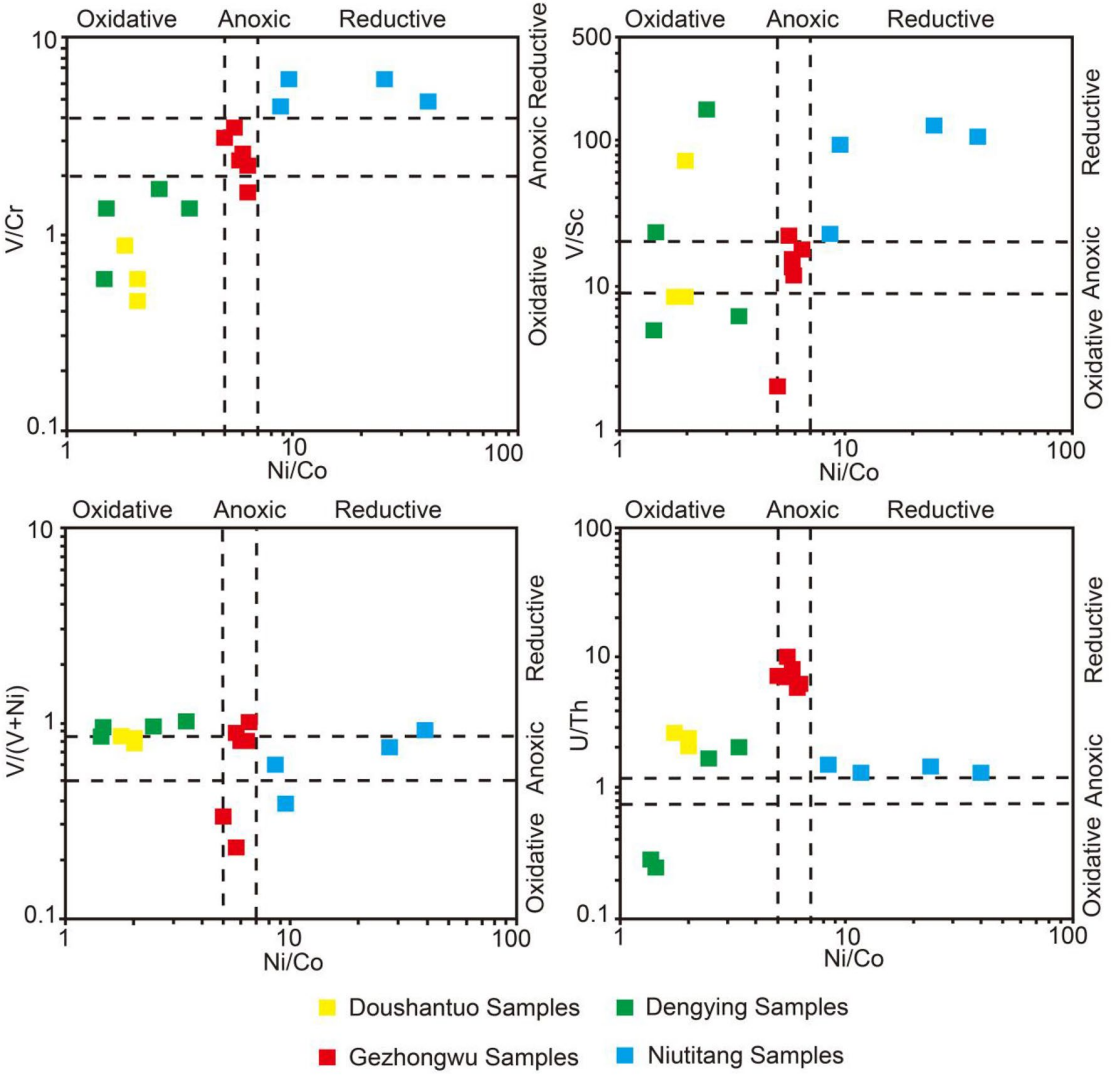
The redox index of samples from the Zhijin and Weng’an phosphorite deposits.

Geochemical indices, including U/Th, Ni/Co, V/(V + Ni), V/Cr, and V/Sc, are used in this study to reconstruct the redox environment during which the phosphorites were formed (Fig. 13). The different indices uncovered similar results: the samples from the Doushantuo Formation and Dengying Formation plot in the oxidizing environment region, the samples from the Gezhongwu Formation plot in the anoxic environment region, and the samples from the Niutitang Formation plot in the reducing environment region. These results indicate that the carbonaceous shale in the Niutitang Formation formed in a reducing environment and that the phosphorite in the Gezhongwu Formation formed in an anoxic environment. However, the dolomite of the Dengying Formation formed in an oxidizing environment, similar to the phosphorite of the Doushantuo Formation from the Weng’an deposit.

**Figure 14:**
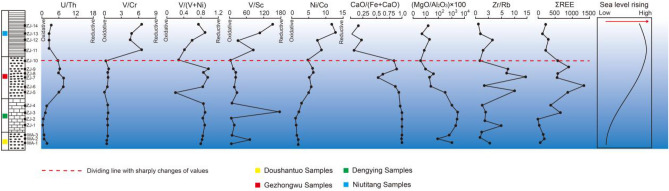
Vertical distribution of the redox index and sea-level variation trend.

The ratios of U/Th, Ni/Co, V/(V + Ni), V/Cr and V/Sc on the chronological profile increase from the bottom to the top (Fig. 14), indicating the change in the hypoxic sedimentary environment from older to younger strata. The Doushantuo and Dengying Formations at the bottom formed in an oxidizing environment. However, the phosphorite of the Gezhongwu Formation shows a distinct weak oxidizing environment. The red dotted line in Fig. 14 marks significant changes in values from the Gezhongwu Formation to the Niutitang Formation, indicating that the oxygen content decreased sharply toward the Niutitang Formation. The carbonaceous shale of the Niutitang Formation developed in an anoxic environment. It should be noted that the results from the vertical variation in V/(V + Ni) and V/Cr are not completely consistent with those drawn from U/Th, Ni/Co and V/Sc in some strata, which may be related to the different enrichment mechanisms of these elements, such as diverse adsorption by organic matter, different sedimentation rates or postdeposition diagenesis, during which changes in the element contents and occurrence forms may occur. Despite multiple solutions due to the different indices, the overall sedimentary environment from the Doushantuo Formation to the Dengying Formation is from oxidation to reduction, combined with the variation in δCe.

### Seawater hydrodynamic analysis

Both the Zhjin and Weng’an phosphorite deposits formed in marine environments, according to the (MgO/Al_2_O_3_) × 100 index (freshwater environment: < 1; sea–land transitional environment: 1–10; seawater sedimentary environment: > 10)^39^. All of the samples in this study have (MgO/Al_2_O_3_) × 100 > 10. However, the study area may experience changes between shallow sea and bathyal marine environments (Fig. 14). Generally, the seawater depth in the coastal zone is closely related to tectonic activity and sea level changes caused by transgression or regression^40^. The marginal shallow sea has lower salinity for the acceptance of freshwater from continental rivers. Moreover, the salinity in the near sea surface increases with the evaporation of seawater; therefore, the salinity of seawater is an important agent indicating variation in the seawater depth^41^. In addition, a deeper seawater depth means weaker dynamic conditions, and vice versa. In this sense, paleosalinity can also be used to reveal the paleoceanographic hydrodynamic environment.

The paleosalinity index of CaO/(Fe + CaO) (CaO/(Fe + CaO) < 0.2) represents low salinity, the ratio of 0.2–0.5 represents medium salinity, and high salinity is determined when CaO/(Fe + CaO) > 0.5^42^. The usage of the above index indicates that paleosalinity varied in different geological periods. The older strata, including phosphorite from the Doushantuo Formation and dolomite from the Dengying Formation, show high paleosalinity, with CaO/(Fe + CaO) > 0.5. The phosphorite from the Gezhongwu Formation is characterized by fluctuations in CaO/(Fe + CaO) varying from 0.16 to 0.90, with an average ratio of 0.70, which indicates that the paleosalinity in this area varies widely. The CaO/(Fe + CaO) ratio of carbonaceous material from the Niutitang Formation varies from 0.01 to 0.23, indicating low and medium paleosalinities. The ancient seawater salinity in the Guizhou phosphorite area was generally high from the late Sinian to the early Cambrian, while from the Gezhongwu Formation to the Niutitang Formation, the salinity experienced a rapid decline, which may reflect the rapid rise in sea level, the deepening of sea water and other changes in the marine environment at that time. This may indicate the occurrence of special paleoceanographic events, such as violent transgression and regression or upwelling. The black shale unit at the top of the Doushantuo Formation is traditionally interpreted as transgressive deposits formed during sea level rise^43,44^.

The paleoceanographical hydrodynamics can also be revealed with element ratios such as Zr/Rb^45^. The grain size fractionating characteristics may be archived in the Zr/Rb ratio. The grain size sorting process during transport dominates the Zr and Rb abundance because Zr is preferentially enriched in the coarser grain size fraction, while Rb tends to be enriched in the finer grain size fraction. Thus, the Zr/Rb ratio increases with the strength of seawater energy^46^. In this study, dolomite from the Dengying Formation has Zr/Rb values from 0.84 to 10.00, with an average of 3.27, indicating a weak hydrodynamic environment. Zr/Rb in the phosphorite from the Gezhongwu Formation fluctuates greatly, varying from 0.65 to 65.45, with an average of 20.19, indicating a turbulent marine environment. However, both the carbonaceous shale from the Niutitang Formation and phosphorite from the Doushantuo Formation have Zr/Rb ratios of 3.29, varying from 0.88 to 7.49, and 1.34, varying from 0.42 to 2.80, respectively. Zr/Rb ratios indicate that the study area shows generally weak marine hydrodynamics from the late Sinian to early Cambrian, except during the Gezhongwu Formation, which showed a turbulent hydrodynamic environment and then weakened gradually to the Niutitang Formation. The recovery of paleoceanographic hydrodynamics is consistent with the results of petrological analysis of phosphorite from the Gezhongwu Formation. The phosphorite from the Gezhongwu Formation of the Zhijin deposit generally contains parallel bedding and cross-bedding, and some phosphorite contains wavy bedding, indicating that the phosphorite of the Zhijin deposit is subject to stronger hydrodynamic action during sedimentation. At the same time, the particle size of the collophanite in the phosphorite from the Gezhongwu Formation of the Zhijin deposit is larger than that from the Doushantuo Formation of the Weng'an deposit, indicating that the former developed higher textural maturity, which also indicates that the Zhijin phosphorite is subject to stronger mechanical action by seawater under shallower seawater depths than the phosphorite of the Weng'an deposit.

### Tectonic setting analysis

The Zhijin phosphorite deposit in Guizhou is located at the southwestern end of the Central Guizhou Uplift in the Yangtze Craton. Most of the faults are linear ladder-like normal faults with structural characteristics similar to those in the ore-rich areas in the margin of the craton. The paleogeographic environment was a shallow sea with weak oxidation^47^. The Weng'an phosphorite deposit in Guizhou is located in the southeastern margin of the Yangtze Craton. The phosphorite of the early Ediacaran Doushantuo Formation was formed in a deep-water terrigenous marine environment^48^. In addition, the contribution of terrigenous weathering materials to the REEs in the phosphorites increased gradually from the early Ediacaran Doushantuo Formation to the early Cambrian Meishucun Formation^48^. Based on the water depth, the Zhijin phosphorite was subjected to late diagenesis and hydrodynamic actions over a longer time period, which promoted the adsorption of REEs by apatite from ambient seawater or pore water^49^. In contrast, the REE contents of Weng'an phosphorite deposits are much lower. Since the Weng'an phosphorite is part of the early Ediacaran Doushantuo Formation, the seawater was relatively anoxic with less biological activity. It lacked a sufficient supply of REEs and an appropriate depositional environment for accumulation. By comparison, the weakly oxidizing environment in which the Zhijin phosphorite deposit formed might have been an important factor controlling REE enrichment.

Furthermore, the REE-rich Zhijin phosphorite deposit may have been related to volcanic hydrothermal activity. Early Cambrian deep-sea hydrothermal vents have been identified in Zunyi, Guizhou. The major and trace element, REE, and sulfur isotopic compositions all indicate that a hydrothermal eruption occurred in this area in the early Cambrian^50^. These hydrothermal materials migrated to the shallow-water areas in Zhijin through upwelling, which may have caused the REE enrichment of the Zhijin phosphorite. However, the Weng'an phosphorite was formed in the late Ediacaran, i.e., earlier than the hydrothermal vent activity. These may be the reasons for the low REE contents of the Weng’an phosphorites. Oxygen isotope geothermometry also indicates that the REE enrichment of the Zhijin phosphorite is related to higher-temperature hydrothermal fluids^48^.

In summary, the tectonic activity on the craton margin provides a suitable depositional environment and material supply for the formation of REE-rich phosphorite. During tectonic activity, the input of volcanic hydrothermal fluid may have provided REEs for the formation of REE-rich phosphorite. Due to the different depths and periods in which deposition occurred, the redox environments and the contributions of terrigenous weathering materials were different. These factors led to the different REE contents of the phosphorite.

## Conclusion

This study analyzed 14 samples from the Zhijin phosphorite mine, including 4 dolomite samples from the Dengying Formation, 6 phosphorite samples from the Gezhongwu Formation, 4 carbonaceous shale samples from the Niutitang Formation, and 3 phosphorite samples from the Doushantuo Formation in the Weng’an deposit. Based on the mineral analysis, element (including major, trace, and rare earth elements) and Sr–Nd isotope measurements, the material sources related to the hydrothermal activity of REEs, the depositional environment and the related tectonic evolution, which is beneficial to the formation of REE-rich phosphorite, were discussed. In particular, the geochemical differences between phosphorites in the Zhijin and Weng’an phosphorites were compared. We reached the following conclusions:

1. REE contents vary from 27.99 to 1409.50 ppm, with an average of 410.57 ppm. REE contents decrease in different strata in the following sequence: phosphorite from Gezhongwu (801.57 ppm) > dolomite from the Dengying Formation (244.29 ppm) > carbonaceous shale from the Niutitang Formation (197.10 ppm) > phosphorite from the Doushantuo Formation (134.92 ppm). The phosphorite from the Gezhongwu Formation is characterized by relatively rich LREEs and HREEs, significant negative Ce anomalies and positive Eu anomalies. It is noted that the phosphorite from the Doushantuo Formation of the Weng’an deposit has higher phosphorus (contains the highest P_2_O_5_ contents of 36.30% to 37.80%, and the average value is 36.83%) than that from the Gezhongwu Formation (0.88–33.30%, 16.08%). However, the latter contains much higher REEs than the former. Thus, a high grade of phosphorite is not necessarily related to high REEs. The material sources may be the dominating factor controlling the enrichment degree of REEs in the phosphorites during different geological periods.
2. Intermittent submarine hydrothermal activities may be the main material source providing a large amount of REEs locally. The hydrothermal sources of REEs for the phosphorite from the Gezhongwu Formation are confirmed by the elemental geochemical characteristics. The Al/(Al + Fe + Mn) ratio, SiO_2_/Al_2_O_3_ ratio, as well as the δEu, δY, Ba/Sr, and Zn-Ni-Co ternary plots all indicate the impact from hydrothermal activities, and the impact increases from carbonaceous from the Niutitang Formation to phosphorite from the Doushantuo Formation to dolomite from the Dengying Formation and to phosphorite from the Gezhongwu Formation. The impact from hydrothermal and REE contributions continued until the Dengying Formation, which is why the dolomite from the Dengying Formation contains higher REEs than the phosphorite from the Doushantuo Formation.
3. The REE-enriched phosphorite from the Gezhongwu Formation likely precipitated in an anoxic environment, as evidenced by the covariance of U/Th, Ni/Co, V/(V + Ni), V/Cr and V/Sc, as well as δCe. The carbonaceous shale from the Niutitang Formation formed in a reducing environment, whereas dolomite from the Dengying Formation and phosphorite from the Doushantuo Formation formed in an oxidizing environment. These results indicate that the sedimentary environment of the Zhijin deposit tended to be anoxic from the bottom up from the late Sinian to the early Cambrian. The precipitation environment is indicated to covary with salinity and sea level change. The CaO/(Fe + CaO) ratio, (MgO/Al_2_O_3_) × 100 and Zr/Rb all suggested that the salinity decreased distinctly in the early Cambrian Gezhongwu Formation, combined with the rising sea level and deeper sea water; moreover, the enhancement of hydrodynamics was observed. It is then indicated that the change in the marine environment to weakly oxic conditions induced by sea level rise may be the main environmental factor impacting REE enrichment.
4. This study concluded that REEs rich in phosphorite from the Gezhongwu Formation were induced by multiple factors. The volcanic hydrothermal activities, biological processes and strong marine dynamic processes that occurred during the Gezhongwu period in the early Cambrian may all be closely related to the enrichment of REEs in phosphorite.

## Data Availability

The authors confirm that the data supporting the findings of this study are available within the article.
